# A Meta-Analysis of the Relative Risk of Mortality for Type 1 Diabetes Patients Compared to the General Population: Exploring Temporal Changes in Relative Mortality

**DOI:** 10.1371/journal.pone.0113635

**Published:** 2014-11-26

**Authors:** Tom W. C. Lung, Alison J. Hayes, William H. Herman, Lei Si, Andrew J. Palmer, Philip M. Clarke

**Affiliations:** 1 Centre for Health Policy, Programs and Economics, School of Population Health, University of Melbourne, Melbourne, VIC 3053, Australia; 2 Sydney School of Public Health, Edward Ford Building (A27), The University of Sydney, Sydney, NSW 2006, Australia; 3 The Department of Internal Medicine, University of Michigan, Ann Arbor, MI 48105, United States of America; 4 The Menzies Research Institute Tasmania, University of Tasmania, Hobart, TAS 7000, Australia; Middlesex University Dubai, United Arab Emirates

## Abstract

**Aims:**

Type 1 diabetes has been associated with an elevated relative risk (RR) of mortality compared to the general population. To review published studies on the RR of mortality of Type 1 diabetes patients compared to the general population, we conducted a meta-analysis and examined the temporal changes in the RR of mortality over time.

**Methods:**

Systematic review of studies reporting RR of mortality for Type 1 diabetes compared to the general population. We conducted meta-analyses using a DerSimonian and Laird random effects model to obtain the average effect and the distribution of RR estimates. Sub-group meta-analyses and multivariate meta-regression analysis was performed to examine heterogeneity. Summary RR with 95% CIs was calculated using a random-effects model.

**Results:**

26 studies with a total of 88 subpopulations were included in the meta-analysis and overall RR of mortality was 3.82 (95% CI 3.41, 3.4.29) compared to the general population. Observations using data prior to 1971 had a much larger estimated RR (5.80 (95% CI 4.20, 8.01)) when compared to: data between; 1971 and 1980 (5.06 (95% CI 3.44, 7.45)); 1981–90 (3.59 (95% CI 3.15, 4.09)); and those after 1990 (3.11 (95% CI 2.47, 3.91)); suggesting mortality of Type 1 diabetes patients when compared to the general population have been improving over time. Similarly, females (4.54 (95% CI 3.79–5.45)) had a larger RR estimate when compared to males (3.25 (95% CI 2.82–3.73) and the meta-regression found evidence for temporal trends and sex (p<0.01) accounting for heterogeneity between studies.

**Conclusions:**

Type 1 diabetes patients’ mortality has declined at a faster rate than the general population. However, the largest relative improvements have occurred prior to 1990. Emphasis on intensive blood glucose control alongside blood pressure control and statin therapy may translate into further reductions in mortality in coming years.

## Introduction

Prior to the availability of insulin, people who developed diabetes before the age of 30 died within an average of 1.4 years of disease onset [1]. The commercial production of insulin in the 1920 s saw a dramatic decrease in mortality for Type 1 diabetes mellitus patients. Whilst the secular downward trend in mortality among the general population over the twentieth century is well known [2], it has not been as well documented in the type 1 diabetes population. One small study in Bucharest reported the decline in mortality over 6 decades [3], whilst other studies only examine this trend in people with type 1 diabetes over the past decade [3]–[5].

The introduction of blood glucose self-monitoring and the increased awareness of the importance of controlling blood glucose to reduce the onset and progression of diabetic complications following the publication of the Diabetes Complications and Complications Trial (DCCT) [6] have led to an intensification of treatment. This has translated into improved life expectancy for patients with type 1 diabetes [3], [4], however it is unclear whether mortality for Type 1 diabetes patients relative to the general population has reduced over time.

Studies have quantified the increased relative risk (RR) of mortality for people with Type 1 diabetes compared with the general population, but there has been no systematic review to estimate pooled measures of RR or to explore the degree to which these vary across regions and over time. A recent large scale study [7] involving individual participant data from 97 prospective studies was unable to separate people with Type 1 and Type 2 diabetes, and so could only provide overall estimates of relative mortality. Type 1 diabetes is typically diagnosed at young ages, and patients are susceptible over a longer period of time [8] to diabetic complications (e.g. renal disease, neuropathy, macrovascular disease); all of which may elevate mortality.

The purpose of this study was to undertake a systematic review of RR of mortality among Type 1 diabetes patients compared to the general population and to conduct a meta-analysis of the findings. Sub-group meta-analyses and meta-regression were also conducted to explore the extent to which the heterogeneity between studies could be explained by temporal changes in the relative risk of mortality for type 1 diabetes patients relative to the general population.

## Materials and Methods

### Data Sources and Searches

Our systematic review aimed to identify studies that compared mortality of Type 1 diabetes patients to the general population and that reported RRs of mortality or standardised mortality ratios (SMR).

We identified studies for inclusion using a systematic literature search of the electronic databases: MEDLINE, EMBASE, CINAHL, PubMed and Cochrane’s systematic reviews. Other databases that were used: The Health Economic Evaluation Database (HEED); the Digital Theses Database; Google Scholar; the TUFTS CEA register; NHS Economic Evaluation Database and the Health Technology Assessment website. We used Google Scholar and Pubmed to conduct a citation search of identified studies and we also examined the citations list of reported articles to identify further potential studies.

### Study Selection

Inclusion criteria included articles published before April 2012 in English in peer reviewed journals in which study subjects have Type 1 diabetes and the overall mortality (as opposed to complication specific studies) among Type 1 diabetes patients is compared to the general population. The review was limited to human studies. Review articles were excluded after references lists had been searched. Where multiple papers used the same cohort in estimating a RR of mortality, only the one that used the longest follow-up was included into the analysis. Studies generally explicitly identified Type 1 diabetes populations. However, we identified Type 1 diabetes populations if patients were diagnosed under 30 years of age and/or classified as insulin only if the study was not clear in its description of diabetes patients.

### Data Extraction and Quality Assessment

The reported RR estimates and their standard errors were extracted from each study for the meta-analysis. Studies that did not report uncertainty around their RR estimate (through standard error or confidence intervals) were excluded from both the meta-analysis and meta-regression. Multiple observations from some studies [9]–[16] were only included if the estimates used different populations to estimate these observations.

We assessed the quality of studies by completing the Strengthening the Reporting of Observational studies in Epidemiology (STROBE) checklist for cohort studies.

Two independent reviewers examined the abstracts and full texts of all articles to determine whether the inclusion criteria were fulfilled. The reviewers also independently extracted the data for the meta-analysis and meta-regression. A third reviewer was assigned to re-examine all full text articles to ensure all data extracted was correct and finalise any discrepancies between the first two reviewers.

### Data Synthesis and Analysis

A meta-analysis was conducted for the RR of mortality for Type 1 diabetes mellitus patients, which pools the results gathered from multiple studies into an overall average effect size. We used the DerSimonian and Laird random effects model for the meta-analysis, which accounts for both the within study variation and expected between study heterogeneity and was conducted in Stata version 12.0 (Stata Corporation, College Station, TX, USA). Studies that measured multiple RRs for different population groups were used as separate observations. We conducted a trim-and-fill analysis to explore the potential of publication bias from our studies.

To explore the possible reasons for heterogeneity among studies numerous sub-group meta-analyses were conducted along with a multivariate meta-regression. Fixed-effects inverse variance meta-analyses were conducted to explore heterogeneity across the categorical groups of studies. Study characteristics extracted for sub-group analysis and meta-regression included:

- A binary variable comparing studies conducted in European countries to non-European countries
- A continuous variable for the year of the median study date in estimating the relative risk of mortality
- A binary variable for sex
- A continuous variable for the sample size of the study
- A binary variable comparing patients’ age at diagnosis to be less than 18 years to patients older than 18 years.

The sub-group meta-analyses were conducted for:

- Sex
- Studies conducted in Europe, in non-European countries and in the United Kingdom
- Studies which used a dataset commencing before 1971, between 1971–80, between 1981–1990, and after 1990
- Studies including patients who were diagnosed before 18 years of age only and studies including patients who were diagnosed after 18 years of age

## Results and Discussion

### Literature search and study characteristics

**Figure 1:**
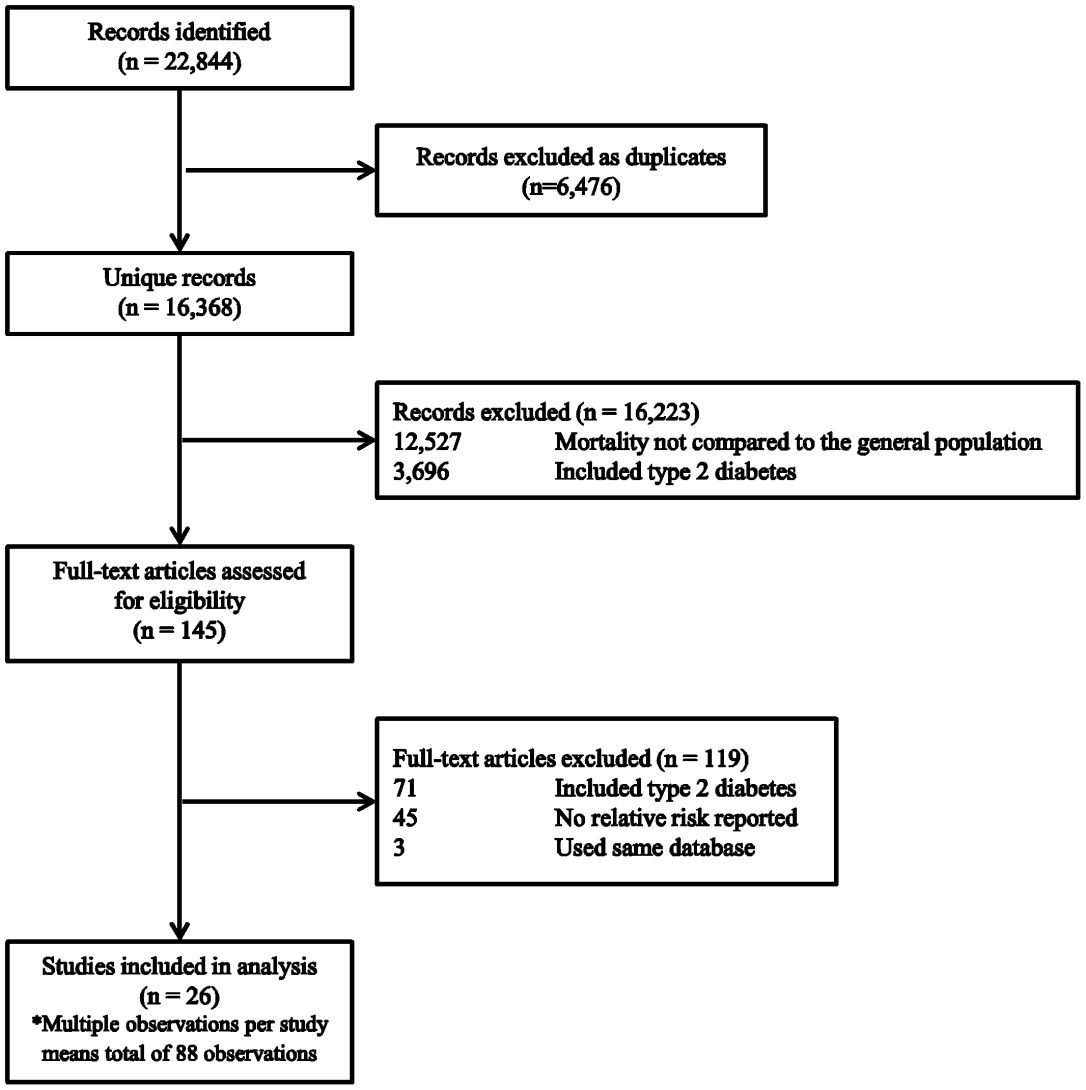
Flow chart of study selection process.

A flow chart of the literature search and its results are reported in Figure 1. Initially, the search identified 22,844 titles and abstracts from all databases. 145 studies were reviewed in full, of which 39 studies were initially included. However, some studies provided more than one RR estimate. Studies [10], [17], [18] and certain observations of studies [19] were excluded if they used the same database as other studies, with a shorter follow-up period. Only 2 studies were different between the first two reviewers, which the third reviewer finalised the discrepancies after discussing the issue between the reviewers.

The meta-analysis and meta-regression used 88 observations from 26 different studies [5], [9], [12], [14], [16], [19]–[40]. Only 1 study estimated RR using hazard ratios [37] and all other studies used SMRs. Details of the studies used, summary statistics and meta-analysis results are shown in Table 1. Studies were conducted in 20 different countries, and the number of patients ranged from 75 to 12,684 per study, with a median of 382 patients. Patient age ranged between 1–79 years, with the majority diagnosed before 18 years. Full search terms of the database MEDLINE and the STROBE checklist for all studies included in the analysis can be found in the electronic supplementary material (Table S1 & Table S2 in File S1).

**Table 1:** List of studies used in meta-analysis, country of analysis, date of conducted studies, age at diagnosis, mean age patients, number of patients in the study, percentage of males in study, years of follow-up, mortality rates and measurement of risk of mortality.

Study	Country	Study period	Age at diagnosis	n	Mortality Rates (95% CI)	Type of Study	Relative Risk Type	Sex
Alleman et al. 2009 [22]	Switzerland	1974–2006	35–54	104	3.80 (3.00–4.80)	Cohort	SMR	Male
Alleman et al. 2009 [22]	Switzerland	1974–2006	35–54	174	5.40 (4.30–6.80)	Cohort	SMR	Female
Asao et al. 2003 [9]	Finland	1965–1979	<18	2817	3.20 (2.80–3.70)	Cohort	SMR	Male
Asao et al. 2003 [9]	Finland	1965–1979	<18	2309	5.20 (4.20–6.30)	Cohort	SMR	Female
Asao et al. 2003 [9]	Japan	1965–1979	<18	566	9.00 (6.80–11.70)	Cohort	SMR	Male
Asao et al. 2003 [9]	Japan	1965–1979	<18	842	18.50 (14.70–23.00)	Cohort	SMR	Female
Barcelo et al. 2007 [23]	Cuba	1965–1970	<10	259	8.58 (6.18–12.28)	Cohort	SMR	Male
Barcelo et al. 2007 [23]	Cuba	1965–1970	<10	245	8.82 (6.70–12.74)	Cohort	SMR	Female
Barcelo et al. 2007 [23]	United States	1965–1970	<10	449	3.42 (2.36–5.13)	Cohort	SMR	Male
Barcelo et al. 2007 [23]	United States	1965–1970	<10	438	3.78 (2.66–5.56)	Cohort	SMR	Female
Botha et al. 1992 [24]	UK and Ireland	1972–1981	<2	179	2.30 (0.60–9.50)	Cohort	SMR	Male
Botha et al. 1992 [24]	UK and Ireland	1972–1981	<2	131	9.90 (4.10–24.20)	Cohort	SMR	Female
Bruno et al. 2009 [25]	Italy	1974–2000	<30	688	1.71 (0.99–2.95)	Cohort	SMR	Male
Bruno et al. 2009 [25]	Italy	1974–2000	<30	522	2.86 (1.29–6.37)	Cohort	SMR	Female
Collado-mesa et al. 1997 [26]	Cuba	1965–1991	<15	259	7.50 (5.30–10.30)	Cohort	SMR	Male
Collado-mesa et al. 1997 [26]	Cuba	1965–1991	<15	245	10.00 (6.90–14.60)	Cohort	SMR	Female
Feltbower et al. 2008 [27]	England	1978–2004	15–29	568	5.80 (3.80–8.60)	Cohort	SMR	Male
Feltbower et al. 2008 [27]	England	1978–2004	< = 14	1742	4.20 (3.20–5.50)	Cohort	SMR	Male
Feltbower et al. 2008 [27]	England	1978–2004	15–29	329	7.50 (3.40–14.30)	Cohort	SMR	Female
Feltbower et al. 2008 [27]	England	1978–2004	< = 14	1607	4.10 (2.60–6.30)	Cohort	SMR	Female
Florkowski et al. 2003 [12]	New Zealand	1984–1999	<30	206	3.00 (2.20–4.00)	Cohort	SMR	Male
Florkowski et al. 2003 [12]	New Zealand	1984–1999	> = 30	NA	1.60 (1.30–1.90)	Cohort	SMR	Male
Florkowski et al. 2003 [12]	New Zealand	1984–1999	<30	229	2.70 (1.90–3.90)	Cohort	SMR	Female
Florkowski et al. 2003 [12]	New Zealand	1984–1999	> = 30	NA	1.70 (1.40–2.00)	Cohort	SMR	Female
Gnavi et al. 2004 [28]	Italy	1991–1999	> = 20	NA	1.98 (1.56–2.47)	Cohort	SMR	Male
Gnavi et al. 2004 [28]	Italy	1991–1999	> = 20	NA	3.36 (2.59–4.28)	Cohort	SMR	Female
Grausland 2010 [29]	Denmark	1973–2006	NA	NA	3.20 (2.80–3.70)	Cohort	SMR	Male
Grausland 2010 [29]	Denmark	1973–2006	NA	NA	3.50 (3.00–4.10)	Cohort	SMR	Female
Harjutsalo et al. 2011 [30]	Finland	1970–1999	< = 14	NA	3.00 (2.70–3.40)	Cohort	SMR	Male
Harjutsalo et al. 2011 [30]	Finland	1970–1999	15–29	93,559	2.60 (2.04–2.80)	Cohort	SMR	Male
Harjutsalo et al. 2011 [30]	Finland	1970–1999	< = 14	NA	5.50 (4.80–6.30)	Cohort	SMR	Female
Harjutsalo et al. 2011 [30]	Finland	1970–1999	15–29	56,345	3.70 (3.20–4.30)	Cohort	SMR	Female
Joner & Patrick 1991 [17]	Norway	1973–1988	< = 14	1,040	2.15 (1.20–3.55)	Cohort	SMR	Male
Joner & Patrick 1991 [17]	Norway	1973–1988	< = 14	874	1.86 (0.60–4.34)	Cohort	SMR	Female
Laing et al. 1999 [31]	United Kingdom	1972–1997	<30	12,684	2.70 (2.50–2.90)	Cohort	SMR	Male
Laing et al. 1999 [31]	United Kingdom	1972–1997	<30	11,047	4.00 (3.60–4.40)	Cohort	SMR	Female
McNally et al. 1995 [14]	England	1940–1949	<17	463	9.44 (4.91–18.14)	Cohort	SMR	Male
McNally et al. 1995 [14]	England	1950–1959	<17	463	6.12 (3.18–11.75)	Cohort	SMR	Male
McNally et al. 1995 [14]	England	1960–1969	<17	463	2.99 (1.34–6.65)	Cohort	SMR	Male
McNally et al. 1995 [14]	England	1970–1979	<17	463	2.30 (0.87–6.10)	Cohort	SMR	Male
McNally et al. 1995 [14]	England	1980–1989	<17	463	1.69 (0.24–11.96)	Cohort	SMR	Male
McNally et al. 1995 [14]	England	1940–1949	<17	382	10.55 (4.41–25.22)	Cohort	SMR	Female
McNally et al. 1995 [14]	England	1950–1959	<17	382	2.86 (0.71–11.41)	Cohort	SMR	Female
McNally et al. 1995 [14]	England	1960–1969	<17	382	5.91 (1.91–18.31)	Cohort	SMR	Female
McNally et al. 1995 [14]	England	1970–1979	<17	382	4.78 (1.82–12.60)	Cohort	SMR	Female
McNally et al. 1995 [14]	England	1980–1989	<17	382	4.05 (0.57–28.54)	Cohort	SMR	Female
Morrish et al. 2001 [21]	Croatia	1975–1988	NA	222	3.46 (2.14–5.28)	Cohort	SMR	Male
Morrish et al. 2001 [21]	Croatia	1975–1988	NA	180	3.36 (1.09–7.84)	Cohort	SMR	Female
Morrish et al. 2001 [21]	Cuba	1975–1988	NA	258	6.85 (4.06–10.82)	Cohort	SMR	Male
Morrish et al. 2001 [21]	Cuba	1975–1988	NA	257	7.90 (3.79–14.53)	Cohort	SMR	Female
Morrish et al. 2001 [21]	Germany	1975–1988	NA	285	6.82 (4.95–9.15)	Cohort	SMR	Male
Morrish et al. 2001 [21]	Germany	1975–1988	NA	275	6.55 (4.35–9.46)	Cohort	SMR	Female
Morrish et al. 2001 [21]	Hong Kong	1975–1988	NA	198	3.44 (1.65–6.32)	Cohort	SMR	Male
Morrish et al. 2001 [21]	Hong Kong	1975–1988	NA	224	6.37 (2.75–12.55)	Cohort	SMR	Female
Morrish et al. 2001 [21]	Poland	1975–1988	NA	241	4.27 (3.17–5.62)	Cohort	SMR	Male
Morrish et al. 2001 [21]	Poland	1975–1988	NA	245	7.00 (4.93–9.64)	Cohort	SMR	Female
Morrish et al. 2001 [21]	Switzerland	1975–1988	NA	278	3.92 (2.53–5.78)	Cohort	SMR	Male
Morrish et al. 2001 [21]	Switzerland	1975–1988	NA	256	7.42 (4.89–10.80)	Cohort	SMR	Female
Morrish et al. 2001 [21]	United Kingdom	1975–1988	NA	254	1.88 (1.13–2.94)	Cohort	SMR	Male
Morrish et al. 2001 [21]	United Kingdom	1975–1988	NA	243	3.38 (2.14–5.07)	Cohort	SMR	Female
Muggeo et al. 1995 [32]	Italy	1986–1991	<18	95	2.70 (0.54–7.90)	Cohort	SMR	Male
Muggeo et al. 1995 [32]	Italy	1986–1991	<18	75	5.08 (1.02–14.86)	Cohort	SMR	Female
Nishimura et al. 2001 [5]	United States	1965–1999	<18	558	3.25 (2.55–3.98)	RCT	SMR	Male
Nishimura et al. 2001 [5]	United States	1965–1999	<18	517	6.90 (5.51–8.46)	RCT	SMR	Female
Podar et al. 2000 [16]	Estonia	1980–1995	<30	269	4.41 (1.79–8.15)	Cohort	SMR	Male
Podar et al. 2000 [16]	Estonia	1980–1995	<30	249	4.86 (1.32–12.44)	Cohort	SMR	Female
Podar et al. 2000 [16]	Finland	1980–1995	<30	2,798	1.38 (0.84–2.13)	Cohort	SMR	Male
Podar et al. 2000 [16]	Finland	1980–1995	<30	2,358	2.26 (1.17–3.95)	Cohort	SMR	Female
Podar et al. 2000 [16]	Lithuania	1983–1995	<30	360	5.28 (2.73–9.22)	Cohort	SMR	Male
Podar et al. 2000 [16]	Lithuania	1983–1995	<30	338	12.56 (6.69–21.47)	Cohort	SMR	Female
Raymond et al. 1995 [33]	United Kingdom	1983–1992	Dec-94	2,482	1.49 (1.29–1.71)	Cohort	SMR	Male
Raymond et al. 1995 [33]	United Kingdom	1983–1992	Dec-94	2,088	1.77 (1.51–2.06)	Cohort	SMR	Female
Riley et al. 1995 [34]	Australia	1984–1993	<30	263	3.20 (2.10–4.70)	Cohort	SMR	Male
Riley et al. 1995 [34]	Australia	1984–1993	> = 30	186	1.40 (1.10–1.80)	Cohort	SMR	Male
Riley et al. 1995 [34]	Australia	1984–1993	<30	217	7.30 (4.60–10.90)	Cohort	SMR	Female
Riley et al. 1995 [34]	Australia	1984–1993	> = 30	169	2.20 (1.70–2.80)	Cohort	SMR	Female
Roberts et al. 2004 [35]	United Kingdom	1968–1996	<30	2,603	6.50 (4.40–9.00)	Cohort	SMR	Male
Roberts et al. 2004 [35]	United Kingdom	1968–1996	<30	2,389	12.80 (8.50–17.90)	Cohort	SMR	Female
Sartor & Dahlquist 1995 [36]	Sweden	1977–1990	<15	2,653	2.62 (1.72–4.00)	Cohort	SMR	Male
Sartor & Dahlquist 1995 [36]	Sweden	1977–1990	<15	2,341	3.84 (2.32–6.35)	Cohort	SMR	Female
Skrivarhaug et al. 2006 [37]	Norway	1973–2002	<15	1,034	3.90 (3.10–4.90)	Cohort	SMR	Male
Skrivarhaug et al. 2006 [37]	Norway	1973–2002	<15	872	4.00 (2.70–5.60)	Cohort	SMR	Female
Soedamah-Muthu et al. 2006 [38]	United Kingdom	1992–1999	< = 35	4,216	3.30 (2.70–4.00)	Cohort	HR	Male
Soedamah-Muthu et al. 2006 [38]	United Kingdom	1992–1999	< = 35	3,497	4.50 (3.50–5.60)	Cohort	HR	Female
Swerdlow & Jones 1995 [39]	United Kingdom	1966–1992	NA	2,907	1.58 (1.50–1.66)	Cohort	SMR	Male
Swerdlow & Jones 1995 [39]	United Kingdom	1966–1992	NA	2,874	2.31 (2.20–2.43)	Cohort	SMR	Female
Waernbaum et al. 2006 [40]	Sweden	1983–1999	15–34	NA	1.90 (1.50–2.40)	Cohort	SMR	Male
Waernbaum et al. 2006 [40]	Sweden	1983–1999	15–34	NA	1.60 (1.00–2.60)	Cohort	SMR	Female
Wibell et al. 2001 [41]	Sweden	1983–1992	15–34	NA	2.10 (1.30–3.10)	Cohort	SMR	Male
Wibell et al. 2001 [41]	Sweden	1983–1999	15–34	NA	1.50 (0.70–3.60)	Cohort	SMR	Female

### Overall meta-analysis

**Figure 2:**
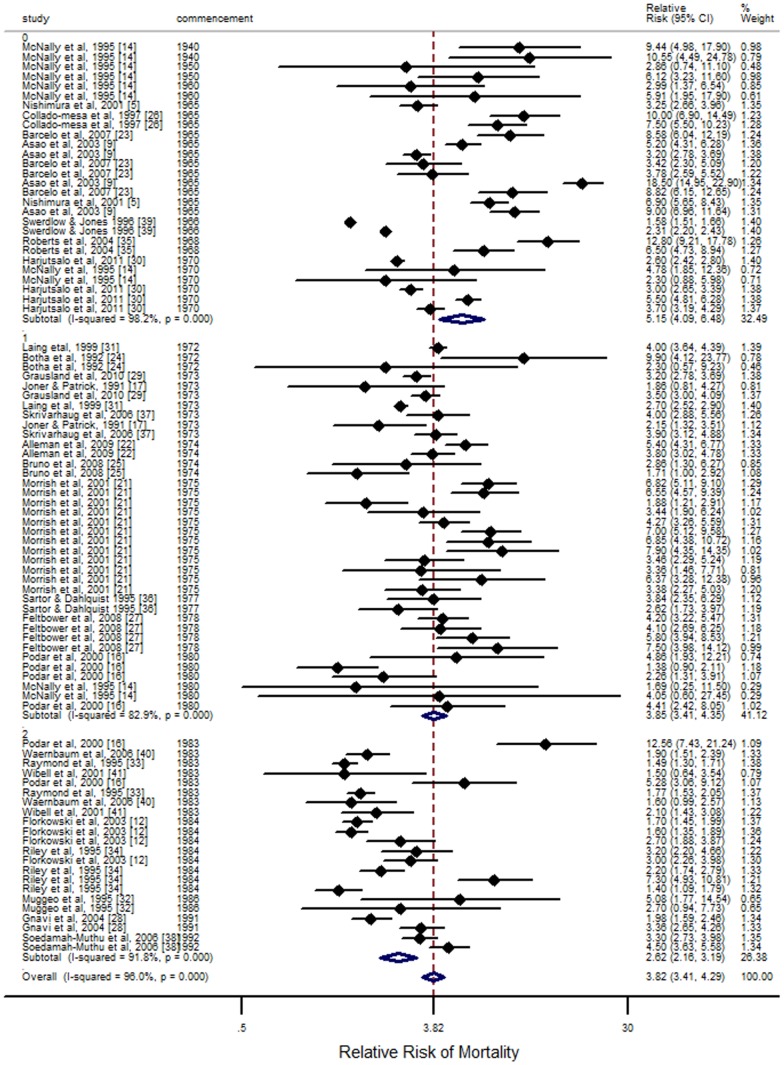
Random effects meta-analysis by median study date (< = 1970, 1971–1980, 1981–1990, >1990). Horizontal bars and circles widths denote 95% CIs, and box sizes indicate relative weight in the analysis.

Results of the overall meta-analysis of 88 observations are shown in Table 2, and a meta-analysis forest plot is depicted in Figure 2, stratified by a studies commencement date. Overall RR of mortality was estimated at 3.82 (95% CI 3.41, 4.29) for Type 1 diabetes mellitus patients compared to the general population. The I-squared value was estimated at 96.0%, indicating the high degree of heterogeneity across studies.

**Table 2:** Meta-analysis results by different categories, showing the number observations used, the pooled estimate and 95% confidence intervals and I^2^ estimate for heterogeneity.

	No. of studies	Pooled Estimate (95% CI)	I^2^ estimate
All studies	88	3.82 (3.41–4.29)	96.0%
Studies commenced before 1970	10	5.80 (4.20–8.01)	69.5%
Studies commenced between 1971–1980	12	5.06 (3.44–7.45)	98.8%
Studies commenced between 1981–1990	50	3.59 (3.15–4.09)	93.1%
Studies commenced after 1990	16	3.11 (2.47–3.91)	92.7%
Studies with patients age at diagnosisbefore 18 years	41	4.93 (4.13–5.88)	90.8%
Studies with patients age at diagnosisafter 18 years	8	2.41 (1.75–3.32)	94.7%
Male	44	3.25 (2.82–3.73)	95.3%
Female	44	4.54 (3.79–5.45)	95.8%
United Kingdom studies	28	3.78 (3.13–4.57)	96.4%
European studies	66	3.56 (3.16–4.00)	95.2%
Non-European studies	22	4.63 (3.28–6.55)	97.0%

### Subgroup analyses and meta-regression analysis to explore cross-study heterogeneity

All sub-group meta-analyses results are also shown in Table 2. Fixed-effects inverse variance meta-analysis for all sub-groups found strong evidence of heterogeneity within sub-groups, and heterogeneity between sub-groups (p<0.01). When conducting separate meta-analyses for men and women, RRs of death were 3.25 (95% CI 2.82, 3.73) and 4.54 (95% CI 3.79, 5.45), respectively. I-squared estimates of 95.3% (men) and 95.8% (women) showed similar heterogeneity as the overall meta-analysis results which suggest that heterogeneity between studies is not explained by gender differences. Similar results were shown for studies conducted in Europe and in the United Kingdom, with RR of mortality of 3.56 (95% 3.16, 4.00) and 3.78 (95% CI 3.13, 4.57), respectively. When stratified by the median study date, observations that used data prior to 1971 had a much higher estimated RR (5.80 (95% CI 4.20, 8.01)) than studies that used data after 1990 (3.11 (95% CI 2.47, 3.92)) with a clear temporal improvement in relative mortality over time. A large amount of heterogeneity can be explained from observations that used data prior to 1971, with an I-squared estimated at 69.5%, compared to 92.7% for studies after 1990.

Further investigation of the possible sources of between-study heterogeneity was performed through multivariate meta-regression analyses and reported in Table 3. 24 studies and 76 observations were included into the primary meta-regression. We decided a linear temporal trend was a better fit than non-linear trends, based on adjusted R-squared values. The assumed linear temporal trend captured by the standardized median date of study was significant, suggesting an improving trend in relative mortality over time for studies whose median date was after 1982. By way of interpretation for every year increase in the year of the data used after 1982, the average RR of mortality decreased by 2%. Similarly, males had a lower RR of mortality on average by 30% than females. Studies conducted in Europe and the numbers of patients in studies were found to have an insignificant effect on the average RR of mortality in the meta-regression. When we included a binary variable to determine whether age at diagnosis was at childhood (<18 years) in a separate meta-regression, it reduced our analysis to 20 studies and 56 observations shown in Table 3. The newly introduced variable was found to have an insignificant effect. However, the adjusted R-squared estimates which explain the proportion of between-study variance improved from 21.8% to 25.74%, respectively.

**Table 3:** Meta-regression results, determining the factors that account for the heterogeneity between different studies’ relative risk of mortality estimates.

24 studies, 74 observations					20 studies, 56 observations				
Variable	Coefficient	P value	95%CI Lower	95%CI Upper	Variable	Coefficient	P value	95%CI Lower	95%CI Upper
Intercept	5.09	<0.01	3.81	6.80	Intercept	6.05	<0.01	4.45	8.21
Median date of study*	0.98	<0.01	0.97	0.99	Median date of study*	0.98	0.03	0.97	0.99
Sex (Male = 1)	0.70	0.01	0.54	0.90	Sex (Male = 1)	0.68	0.01	0.50	0.91
Region (Europe** = 1)	0.80	0.13	0.60	1.07	Region (Europe** = 1)	0.88	0.43	0.63	1.22
Number of patients in study***	0.99	0.34	0.99	1.00	Number of patients in study***	0.99	0.35	0.99	1.00
					Age at diagnosis (>18 years old = 1)	0.66	0.13	0.38	1.13

*Median date of study standardized by deducting the median date of studies of 1982.

**66 observations conducted in Europe.

***Number of patients in study standardized by deducting the mean number of patients of 1163.

The trim-and-fill analysis plot is reported in Figure S2 in File S1. No trimming of data has been performed, suggesting there is no evidence of publication bias.

## Discussion

This study represents the first meta-analysis of the RRs of mortality for Type 1 diabetes patients compared to the general population. Although the meta-analysis showed on average over three-fold RR in mortality compared to the general population, there is a clear reduction in the risk of mortality when examining populations over time. Our sub-group analyses showed studies examining databases prior to 1971 had almost double the RR of mortality relative to the general population when compared to studies using databases after 1990. Whilst the general population’s mortality has reduced during this period, it is clear that for type 1 diabetes patients, their mortality has been declining at an even faster rate than the general population.

To put this into perspective, a recent study estimated SMR in the months following an acute myocardial infarction to be four times greater which is similar to the overall relative mortality in our analysis across all studies [41]. As we have demonstrated above there is a significant decline in relative mortality. The pooled standardised mortality of studies initiated since 1990 suggests around a three-fold all-cause mortality risk. While this suggests significant improvements in relative survival of people with Type 1 diabetes, they are still at a greater mortality risk than people with Type 2 diabetes [42], or long-term survivors of a myocardial infarction [41].

Our results also place in context two large studies estimating relative mortality of people with Type 1 diabetes based on registries in Denmark [43] and Scotland [44]. While Jorgensen et al. [43] reported improvements in mortality rates in type 1 diabetes patients over the study period between 2002 and 2011, the relative risks of 2.58 (95% CI 2.23, 2.98) for men and 2.71 (95% CI 2.18, 3.38) reported in the Scottish study based on data collected between 2005 and 2007 [44] are not significantly different from our estimates of all studies initiated since 1980.

While we have observed secular improvement in relative mortality for people with type 1 diabetes for over 50 years, the largest relative improvements occurred prior to 1980. However, it is important to continue to monitor the relative mortality of people with type 1 diabetes, particularly in light of published results from the DCCT [6] which indicate metabolic memory and legacy effects from intensive blood glucose control on complications of diabetes [45], [46] which may translate into further improvements in mortality in coming years.

As expected there was a large amount of between-study heterogeneity. Differences between the composition of Type 1 diabetes cohorts in the study and the associated background population with which to estimate RR of mortality from along with studies being conducted at different time periods leads one to expect a certain level of heterogeneity between studies. We conducted a number of sub-group meta-analyses (European studies, non-European studies, stratification by commencement date of studies and sex) and still found high levels of heterogeneity, as represented by the large I-squared values in Table 2. A random-effects meta-regression was conducted to combine the results of studies to account for the variation. The meta-regression shows a negative linear trend, with a coefficient of 0.98 (95% CI 0.97–0.99) suggesting decreasing relative mortality over time. The only other significant factor that explained the variation between studies was by sex as women were estimated to have a higher RR of mortality when compared to men, 4.54 (95% CI 3.79, 5.45) and 3.25 (95% CI 2.82, 3.73) respectively. This is indicative of the improving level of treatments for patients with Type 1 diabetes. However, there were still unobserved factors which attributed to heterogeneity after conducting a meta-regression and sub-group meta-analyses. Differences in the duration of studies and in the baseline characteristics of the type 1 diabetes patients, the background mortality of the general population to which the RR are calculated and the inconsistent reporting of patient characteristics across studies meant the influence of other confounders (diabetes-specific complications, duration of diabetes, median age of diagnosis, body weight, smoking, type of treatment regimen, HbA1c levels, etc.) that potentially could be associated with heterogeneity surrounding RR in mortality of Type 1 diabetes patients could not be assessed. Previous studies examining SMR’s in a meta-analysis framework also reported high levels of between-study heterogeneity [47], [48].

There was a considerable range of estimated RR of mortality, with the lowest estimated at 1.38 (95% CI 0.84–2.13) by a study in Finland [16] and the highest RR of mortality estimated at 18.5 (95% CI 14.7–23.0) in Japan [9]. Whilst this meta-analysis shows considerable heterogeneity in RR of mortality between different developed countries, there is a lack of information reported in less developed countries, and studies reporting mortality values were often based on a small number of cases [49]. Further research needs to be conducted in order to determine the RR of mortality in developed countries compared to developing countries.

## Conclusions

In conclusion, this study has estimated people with type 1 diabetes have an elevated risk of mortality when compared to the general population, although the gap between the two populations have been decreasing. However, the sub-group meta-analyses suggests that the largest reductions in relative mortality have been achieved prior to 1980, although improvements in treatments following the DCCT imply metabolic memory and legacy effects of intensive glucose control along with statin treatment and blood pressure control could potentially reduce relative mortality further in the future.

## Supporting Information

File S1 **Tables S1 and S2 and Figures S1 and S2.** Table S1. Summary of terms used in the Medline search strategy. Table S2. STROBE checklist for cohort studies. Figure S1. Cumulative meta-analysis by median study date (< = 1970, 1971–1980, 1981–1990,>1990). Horizontal bars and circles widths denote 95% CIs, and box sizes indicate relative weight in the analysis. Figure S2. Trim-and-fill analysis of the included estimates. No trimming of data was performed, suggesting no evidence of publication bias.
(DOCX)
