# Supplementary material for: A Meta-Analysis of the Relative Risk of Mortality for Type 1 Diabetes Patients Compared to the General Population: Exploring Temporal Changes in Relative Mortality
Source: PLoS One. 2014 Nov 26;9(11):e113635. doi: 10.1371/journal.pone.0113635 (PMC4245211; doi:10.1371/journal.pone.0113635)
Supplement: File S1 — Tables S1 and S2 and Figures S1 and S2. Table S1. Summary of terms used in the Medline search strategy. Table S2. STROBE checklist for cohort studies. Figure S1. Cumulative meta-analysis by median study date (< = 1970, 1971–1980, 1981–1990,>1990). Horizontal bars and circles widths denote 95% CIs, and box sizes indicate relative weight in the analysis. Figure S2. Trim-and-fill analysis of the included estimates. No trimming of data was performed, suggesting no evidence of publication bias. (DOCX) [file pone.0113635.s001.docx]

**Electronic Supplementary Material**

**Table S1: Summary of terms used in the Medline search strategy**

MESH/Subject Headings

- Diabetes Mellitus, Type 1
- Mortality
- Risk
- Diabetes Complications

Text Words

- All-cause mortality
- Relative Risk*s
- Hazard Ratio*s
- Standardised mortality ratio*s
- Relative mortality
- HR
- SMR
- RR
- Type 1 diabetes
- Mortality rates*s
- Insulin dependent diabetes
- Type 1 diabetes mellitus
- Insulin dependent diabetes mellitus

**Databases searched**

Medline

Cinahl database

Cochrane Database of Systematic Reviews

Google Scholar

Health Technology Assessment (HTA)

Embase

NHS Economic Evaluation Database

**Table S2: STROBE checklist for cohort studies**

# STROBE Checklist:

|  | Item No | Recommendation | Alleman et al, 2009 [22] | Asao et al, 2003 [9] | Barcelo et al, 2007 [23] |  |  |
| --- | --- | --- | --- | --- | --- | --- | --- |
| **Title and abstract** | 1 | (*a*) Indicate the study’s design with a commonly used term in the title or the abstract | Long-term cardiovascular and non-cardiovascular mortality in women and men with type 1 and type 2 diabetes mellitus: A 30-year follow-up in Switzerland | Long-term mortality in nationwide cohorts of childhood-onset type 1 diabetes in Japan and Finland | A cohort analysis of type 1 diabetes mortality in Havana and Allegheny County, Pittsburgh, PA |  |  |
|  |  | (*b*) Provide in the abstract an informative and balanced summary of what was done and what was found | BACKGROUND: While studies from other countries have shown an excess mortality in diabetic individuals when compared with the general population, comparable long-term data is not available for Switzerland. AIMS: To assess gender-specific cardiovascular and non-cardiovascular mortality of patients with type 1 and type 2 diabetes compared with the general Swiss population between 1974 and 2005. DESIGN: 533 patients (225 type 1, 308 type 2 diabetes, 52.2% men) were followed for 30 years (10349 person-years). RESULTS: Diabetic patients had an increased all-cause mortality compared with the general population (SMR [95% CI] 3.8 [3.5-4.3]). Standardised mortality ratio (SMR) was higher for type 1 compared with type 2 diabetic patients (4.5 [3.8-5.3] vs 3.5 [3.1-4.0], p = 0.032). For cardiovascular and non-cardiovascular deaths SMRs were 5.6 (95% CI 4.8-6.6) and 2.7 (2.3-3.1) and did not differ according to type of diabetes. SMRs for all-cause and cardiovascular mortality were significantly higher in women compared with men in type 1 (p <0.05 and p <0.01) and type 2 diabetes (p <0.001 and p <0.01). In both types of diabetes, SMRs significantly decreased during the last two decades (p for trend 0.004 and 0.002). CONCLUSIONS: Patients with type 1 and type 2 diabetes had an increased long-term mortality compared with the general Swiss population. Excess mortality was higher in type 1 compared with type 2 diabetes and in women compared with men for both types of diabetes, but steadily decreased over the last two decades. | OBJECTIVE: This study compares mortality from type 1 diabetes in Japan and Finland and examines the effects of sex, age at diagnosis, and calendar time period of diagnosis on mortality. RESEARCH DESIGN AND METHODS: Patients with type 1 diabetes from Japan (n = 1,408) and Finland (n = 5,126), diagnosed from 1965 through 1979, at age <18 years, were followed until 1994. Mortality was estimated with and without adjustment for that of the general population to assess absolute and relative mortality using Cox proportional hazard models. RESULTS: Overall mortality rates in Japan and Finland were 607 (95% CI 510-718) and 352 (315-393), respectively, per 100,000 person-years; standardized mortality ratios were 12.9 (10.8-15.3) and 3.7 (3.3-4.1), respectively. Absolute mortality was higher for men than for women in Finland, but relative mortality was higher for women than for men in both cohorts. Absolute mortality was higher in both cohorts among those whose diabetes was diagnosed during puberty, but relative mortality did not show any significant difference by age at diagnosis in either cohort. In Japan, both absolute and relative mortality were higher among those whose diagnosis was in the 1960s rather than the 1970s. CONCLUSIONS: Mortality from type 1 diabetes was higher in Japan compared with Finland. The increased risk of death from type 1 diabetes seems to vary by sex, age at diagnosis, and calendar time period of diagnosis. Further investigation, especially on cause-specific mortality, is warranted in the two countries. | OBJECTIVE: To examine the mortality of type 1 diabetes (T1D) in two countries with very different health care systems using two population-based registries of childhood-onset T1D one in Havana (HA), Cuba, and the other in Allegheny County (AC), USA. RESEARCH DESIGN AND METHODS: Cases diagnosed with T1D between 1965 and 1980 in HA and between 1965 and 1979 in AC were included. Follow-up started with diagnosis in each individual and ended as of 1 January 1991, or with death. Life-table analyses were used to examine the mortality rates in both populations by duration of diabetes. RESULTS: Cumulative mortality by January 1991 in HA (14% in males and females, respectively) was higher than in AC (7% in males and 9% in females) for both genders (males, p=0.0005; females, p=0.0491). Mortality rates were considerably higher in HA for both men and women than in AC however, among females confidence intervals overlapped. Overall mortality rate for Caucasians (AC) was significantly lower than that for African-Americans (AC) or Hispanics (HR). An analysis of causes of death showed a greater proportion of deaths attributed to nephropathy (48.6%) in HA while acute complications (36%) and infections (27%) were more frequent in AC. CONCLUSIONS: This study shows a two-fold greater mortality among people with childhood-onset T1D in Havana, Cuba, than in Allegheny, USA. Different strategies may be needed to increase survival among those with type 1 diabetes in the USA and Cuba. |  |  |
| Introduction | | |  |  |  |  |  |
| Background/rationale | 2 | Explain the scientific background and rationale for the investigation being reported | Estimates for excess mortality due to cardiovascular causes in diabetic patients have been shown to vary widely across European countries. Conflicting evidence on the influence of confounders have resulted in studies with different outcomes. | Mortality from childhood-onset type 1 diabetes differs greatly from country to country (1– 4). The Diabetes Epidemiology Research International (DERI) Mortality Study Group has previously shown that Japanese patients with type 1 diabetes diagnosed from 1965 through 1979 had higher mortality rates than patients in three other countries: Israel, Finland, and the U.S. | Although life expectancy for people with type 1 diabetes (T1D) has dramatically increased in the developed world, childhood diabetes is still a threat to life in developing countries. |  |  |
| Objectives | 3 | State specific objectives, including any prespecified hypotheses | Assessment of long-term mortality in type 1 and 2 diabetic patients in Switzerland over 30 years, compared to gen. pop and by gender. | A follow-up study through 1994 of the Japanese and Finnish cohorts of the original DERI study (5,6) to observe how mortality patterns from type 1 diabetes might have changed with the age of the subjects and longer duration of diabetes. | The objective of this study was to further examine mortality in two countries with very different health care systems by comparing long-term outcomes of two population-based registries of childhood-onset T1D, one in Havana (HA), Cuba and the other in Allegheny  County (AC), USA |  |  |
| Methods | | |  |  |  |  |  |
| Study design | 4 | Present key elements of study design early in the paper | Long term large scale comparison across Type 1 and Type 2 diabetic patients in Switzerland. | Long term comparison of type 1 diabetes across Japan and Finland | Long term comparison of type 1 diabetes across USA and Cuba |  |  |
| Setting | 5 | Describe the setting, locations, and relevant dates, including periods of recruitment, exposure, follow-up, and data collection | Swiss participants from “WHO Multinational Study of Vascular in Diabetes”. | Patients with Type 1 Diabetes from Japan and Finland | Patients with type 1 diabetes placed on a daily insulin injection before 15, and a resident of Allegheny Country, USA, or Havana, Cuba. |  |  |
| Participants | 6 | (*a*) Give the eligibility criteria, and the sources and methods of selection of participants. Describe methods of follow-up | 533 Patients; Age 35-54, recruited Feb 1974-Mar 1977.  Considered Type 1 if Insulin needed within 1 year of diagnosis, otherwise Type II. Recruited from 'WHO Multinational Study of Vascular Diabetes'. Follow-up on 1-Jan-06 from population registries. | 533 Patients; Aged 35-43; Switzerland; recruited from Feb-1974 to March-1977; Followed up on 1-Jan-2006; Eligible criteria: Type I if Insulin needed within a year; Type II otherwise; Recruited from 'WHO Multinational Study of Vascular Diabetes' | 504 Cuban patients; 887 US patients; from 1965 to 1980; followed up on 1-Jan-91 with a diagnosis per patient; Diagnosis of Diabetes; daily insulin injections before 15th birthday; resident of registry caption area; recruited from Census and Incidence registry. |  |  |
|  |  | (*b*) For matched studies, give matching criteria and number of exposed and unexposed | Not reported | Not reported | Not reported |  |  |
| Variables | 7 | Clearly define all outcomes, exposures, predictors, potential confounders, and effect modifiers. Give diagnostic criteria, if applicable | Age; Sex | Age; Sex | Age; Sex |  |  |
| Data sources/ measurement | 8* | For each variable of interest, give sources of data and details of methods of assessment (measurement). Describe comparability of assessment methods if there is more than one group | WHO study | WHO study | Incidence registry; contact with paediatricians; census data |  |  |
| Bias | 9 | Describe any efforts to address potential sources of bias | Not reported | None reported | None reported |  |  |
| Study size | 10 | Explain how the study size was arrived at | From WHO study | From nationwide surveys | From study population size |  |  |
| Quantitative variables | 11 | Explain how quantitative variables were handled in the analyses. If applicable, describe which groupings were chosen and why | Not reported | Not reported | Not reported |  |  |
| Statistical methods | 12 | (*a*) Describe all statistical methods, including those used to control for confounding | Kaplan-Meier survival analysis; longrank test for gender within diabetes type; 95% confidence interval assumed death followed Poisson distribution; | Overall and stratified mortality rates calculated by deaths per 100,000 person-years. Standardised mortality stats from WHO. 95% CI's calculated assuming deaths occurred with Byar's approximation for a Poisson distribution | Survival analysis + Cox proportional hazard model |  |  |
|  |  | (*b*) Describe any methods used to examine subgroups and interactions | Not reported | Not reported | Not reported |  |  |
|  |  | (*c*) Explain how missing data were addressed | Not reported | Not reported | Not reported |  |  |
|  |  | (*d*) If applicable, explain how loss to follow-up was addressed | Lost patient 35 removed from study | Removed 22 patient as found to have Down's syndrome | Not reported |  |  |
|  |  | (*e*) Describe any sensitivity analyses | Not reported | Not reported | Not reported |  |  |
| Results | | |  |  |  |  |  |
| Participants | 13* | (a) Report numbers of individuals at each stage of study—eg numbers potentially eligible, examined for eligibility, confirmed eligible, included in the study, completing follow-up, and analysed | 352 patient died during study | 5,126 patients died during study |  |  |  |
|  |  | (b) Give reasons for non-participation at each stage | Death | Death | Death |  |  |
|  |  | (c) Consider use of a flow diagram |  |  |  |  |  |
| Descriptive data | 14* | (a) Give characteristics of study participants (eg demographic, clinical, social) and information on exposures and potential confounders | Swiss patients aged between 35 and 54 who had been diagnosed with diabetes | Japanese and Finnish patients aged <18 yrs when diagnosed with diabetes | US and Cuban patients diagnosed with diabetes type 1 pre 15 yrs old |  |  |
|  |  | (b) Indicate number of participants with missing data for each variable of interest | Not reported | Not reported | Not reported |  |  |
|  |  | (c) Summarise follow-up time (eg, average and total amount) | 30 years | 15 years | 15 years |  |  |
| Outcome data | 15* | Report numbers of outcome events or summary measures over time | 18 outcomes reported | 13 outcomes reported | 16 outcomes reported |  |  |
| Main results | 16 | (*a*) Give unadjusted estimates and, if applicable, confounder-adjusted estimates and their precision (eg, 95% confidence interval). Make clear which confounders were adjusted for and why they were included | Diabetic patients had increased all-cause mortality compared with gen. pop. (SMR [95% CI] 3.8 [3.5-4.3]). SMR higher for Type I cf. Type II (4.5 [3.8-5.3] vs 3.5 [3.1-4.0], p=0.032). For cardiovascular and non-cardivascular deaths SMRs were 5.6 (95% CI 4.8-6.6) and 2.7 (2.3-3.1) and differ according to type of diabetes. SMRs for all-cause and cardiovascular mortality were sig. higher in women cf. men in Type I, and Type II. In both types, SMRs sig. decreased during last two decades. | Risk of death 1.93x higher in men cf. women; 1.62x higher for patient diagnosed in pubertal age, cf. prepubertal age. Absolute mortality rates for Type I Diabetes in Japan almost twice that of Finland | Cox-model adjusted, odds ratio was 3.3x higher for Hispanics than Caucasians; the non-adjusted risk for african-americans for any-cause mortality was 2.8. Mortality rate of nephropathy was higher in HA than AC |  |  |
|  |  | (*b*) Report category boundaries when continuous variables were categorized | Not reported | Not reported | Not reported |  |  |
|  |  | (*c*) If relevant, consider translating estimates of relative risk into absolute risk for a meaningful time period | Not reported | Not reported | Not reported |  |  |
| Other analyses | 17 | Report other analyses done—eg analyses of subgroups and interactions, and sensitivity analyses | Not reported | Not reported | Not reported |  |  |
| Discussion | | |  |  |  |  |  |
| Key results | 18 | Summarise key results with reference to study objectives | See 16 | See 16 | See 16 |  |  |
| Limitations | 19 | Discuss limitations of the study, taking into account sources of potential bias or imprecision. Discuss both direction and magnitude of any potential bias | Study design prevented repetitive collection of specific treatment modalities or clinical parameters during follow up; Absolute numbers small; Swiss gen. pop. Used to calculate SMRs, which could be underestimation of excess risk | However, the finding of a greater relative mortality among women compared  with men needs to be evaluated, especially for cause-specific mortality, since sex differences in the mortality of patients with type 1 diabetes have been inconsistent in previous studies | It was not possible to expand the current analysis to 1999 because of the unavailability of follow-up information for the HA cohort |  |  |
| Interpretation | 20 | Give a cautious overall interpretation of results considering objectives, limitations, multiplicity of analyses, results from similar studies, and other relevant evidence | Increased mortality of diabetic patient Female patient sig. greater mortality rates cf. men | Mortality from type 1diabetes was higher in Japan compared with Finland. This effect seems to vary by sex, age at diagnosis, and calendar time period of diagnosis. From clinical and public health points of view, mortality of the general population should be taken into account when determining both the magnitude of the risk of death related to diabetes and the reasons for the increased risk. | In conclusion, T1D is an uncommon disease in Cuba where incidence rates among children were four to seven times lower than in the USA [26], this may explain our findings showing that mortality rates were much higher in HA particularly in terms of renal disease  where long-term treatment may not be as fully  developed. On the other hand, acute mortality was higher in AC, which may reflect a lower availability of easily accessible basic care particularly among African-Americans than in HA. |  |  |
| Generalisability | 21 | Discuss the generalisability (external validity) of the study results | Not reported | Possible explanations for the adverse effects of diagnosis in pubertal age include the heterogeneity of the etiology of diabetes (35–37) and psychosocial problems during pubertal age. However, recent studies have shown the importance of prepubertal, as well as pubertal and postpubertal, duration of diabetes for diabetic nephropathy and retinopathy (21,22,38). | There have been reports from several developed countries showing a decrease in the risk of dying with time, such as those in Norway [15],  Denmark [16], UK [17,18] and Japan [19]. The same effect is apparent in the current study for AC but there was little difference in mortality in HA with diagnosis between 1965–1970 and 1971–1980. |  |  |
| Other information | | |  |  |  |  |  |
| Funding | 22 | Give the source of funding and the role of the funders for the present study and, if applicable, for the original study on which the present article is based | Not reported | Finnish Academy (Grant Nos. 38387, 46558) | Not reported |  |  |

*Give information separately for exposed and unexposed groups.

|  | Item No | Recommendation | Botha et al, 1992 [24] | Bruno et al, 2008 [25] | Collado-mesa et al, 1997 [26] |  |  |
| --- | --- | --- | --- | --- | --- | --- | --- |
| **Title and abstract** | 1 | (*a*) Indicate the study’s design with a commonly used term in the title or the abstract | Diabetes diagnosed before the age of 2 years: mortality in a British cohort 8-17 years after onset | Short-term mortality risk in children and young adults with type 1 diabetes: the population-based Registry of the Province of Turin, Italy | Mortality of childhood-onset IDDM patients. A cohort study in Havana City Province, Cuba |  |  |
|  |  | (*b*) Provide in the abstract an informative and balanced summary of what was done and what was found | Childhood diabetes diagnosed before the age of 24 months presents specific management problems. We report here on the establishment (using the British Diabetic Association [BDA] Children's Register) and mortality of a cohort of children with diabetes diagnosed before age 24 months. Children registered during the period 1972-1981 were traced by contacting consultants or by using the National Health Service Central Registers (NHSCR) of the Office of Population Censuses and Surveys (OPCS). Standardized mortality ratios (SMR) were estimated using person-years of follow-up for each child and age-specific death rates for the England and Wales population for the years 1972-1989. Of 339 children notified during 1972-1981, 231 were traced through consultants and 99 of the remaining 108 through the NHSCR. Twenty were found to be ineligible. The cohort available for mortality analysis comprised 310 (97%) of 319 eligible children. Their age at the time the cohort was established was 8-18 years, and their duration of diabetes 8-17 years. The male:female ratio is 1.4:1. Of 310 children studied, seven have already died: SMR 5.4 (95% CI: 2.5-11.5). We have established a large, unique cohort of children with diabetes diagnosed before age 24 months and still living in the UK and Ireland. The natural history including mortality and occurrence of complications will be analysed prospectively in this cohort and compared to other cohorts of similar disease duration, but later age at onset. | Short-term mortality risk in young diabetic people is an indicator of quality of care. We assessed this in the Italian incident population-based registry of Turin. The study base included 1210 incident cases (n=677 aged 0-14 years and n=533 aged 15-29 years) with diabetes, onset period 1974-2000 in the Province of Turin, Italy. The relevant timescale for analysis was the time since the onset of diabetes to death, or till 31 December 2003. Standardized mortality ratio (SMR) for all-cause mortality was computed using the Italian population as a standard, by 5 years, age group, sex, and calendar period. Mean attained age of the incident cohort was 29.7 years (range 5.2-49.7 years). During a mean follow-up period of 15.8 years (range 2.0-29.9 years), there were 19 deaths in 15,967. Nine person-years of observation (n=9.5 expected deaths), giving an all-cause mortality rate of 1.19/1000 person-years (95% CI 0.76-1.87) and an SMR of 1.96 (1.25-3.08). In no cases did death occur at the onset of diabetes or in childhood. Out of 19 deaths, 9 were diabetes related (n=6 coma and n=3 end-stage renal disease). In Cox regression analysis, the hazard ratio (HR) was higher in adult-onset than in childhood-onset diabetes (HR=3.90, 95% CI 1.14-13.39), independently of calendar period and gender. (1) Children and young adults with type 1 diabetes experienced a two-fold higher short-term mortality risk than Italian people of similar age and sex and (2) the risk was higher in adult-onset than in childhood-onset diabetes. The quality of diabetes care should be improved to prevent early deaths. | OBJECTIVE: To determine the survival pattern and the underlying cause of death in a cohort of childhood-onset IDDm subjects from Havana City Province, Cuba. RESEARCH DESIGN AND METHODS: This was a descriptive study carried out on a historical cohort of IDDM subjects with disease onset before 15 years of age in Havana City Province, Cuba. The cohort was assembled from several sources. Subjects were diagnosed from 1965 to 1980, and their vital status was assessed at 31 December 1991. Cumulative survival rate was calculated by the Kaplan-Meier method, and a univariate analysis was performed. To test survival differences between groups, the Cox-Mantel test was used. To compare the cohort mortality with the general population, standardized mortality ratios by sex and age were calculated. Specific causes of death were determined by a committee examining death certificates, clinical records, and necropsy reports. RESULTS: A total of 504 subjects were identified, and the mean follow-up time was 17.5 years. Of the subjects, 70 (13.9%) had died at 31 December 1991. Overall, the cohort had a 71% cumulative survival rate at 25 years of IDDM duration. There were no survival differences according to sex or calendar period of IDDM diagnosis. Statistically significant differences were found among age-at-diagnosis groups. The group with a peripubertal age at diagnosis showed the worst prognosis. The cohort experienced 8.5 times the all-causes death rate, compared with the general population. Renal disease accounted for almost half the deaths. CONCLUSIONS: IDDM subjects from Havana City Province, Cuba, showed a better survival pattern than IDDM subjects from other developing countries. However, when compared with IDDM populations from developed countries, there is a survival reserve to be achieved by reducing mortality due to renal disease and infections. |  |  |
| Introduction | | |  |  |  |  |  |
| Background/rationale | 2 | Explain the scientific background and rationale for the investigation being reported | Childhood diabetes diagnosed before the age of 24 months, while forming no more than 6%' (also M A Metcalfe—personal communication) of newly diagnosed childhood diabetes, presents very specific management problems to clinicians and parents alike. Some of the unique features which have been described in small groups of children are male predominance,2"4 a high prevalence of diabetes in the family,3 many episodes of severe hypoglycaemia,35 few episodes of ketoacidosis,3'5 lesser incidence of vascular complications before 30-34 years' duration than childhood diabetes of later onset,' possible long term cognitive difficulties,6"10 and suggestions of early  mortality.1'3 | Epidemiological studies found that the risk of young people with type 1 diabetes dying is 2e3 times higher than non-diabetic people of similar age [1e9]. Whereas after the age of 30 years most deaths are due to chronic complications [7e9], in people aged 30 years mortality is mainly due  to acute diabetes-related events (diabetic ketoacidosis, hypoglycaemia) or to accident and violence [1e6,10]. | Mortality data for cohorts of people with IDDM from developing countries are lacking. This might be due to the relatively low prevalence of the disease and the inevitable problems in identification of subjects and ascertainment of death. |  |  |
| Objectives | 3 | State specific objectives, including any prespecified hypotheses | In this paper we report on the process of establishing a cohort for investigation and on mortality since diagnosis | In this report, we were interested in assessing the short-term impact of type 1 diabetes on survival, by taking advantage of the first Italian incidence registry, recruiting cases up to age 29 years among residents of the Province of Turin since 1984 [15,16]. The aims of our analyses were (1) to estimate short-term mortality in the cohort with disease onset in the period 1974e2000 with respect to the reference Italian population and (2) to assess differences in mortality risk between childhood (0e14 years) and young adult-onset (15e29 years) type 1 diabetes. | Long term follow up study performed on a large cohort of people with IDDM from a developing country. |  |  |
| Methods | | |  |  |  |  |  |
| Study design | 4 | Present key elements of study design early in the paper | The BDA supplied us with 427 records of children notified to the Register during that period as having developed diabetes before the age of 24 months | The population-based cohort of incident cases of the type 1 Diabetes Registry of the Province of Turin enrolled 1053 persons aged 0e29 years from January 1, 1984 to December 31, 2000. | National Registry of IDDM patients, with 504 subjects identified, from 1965 to 1980. |  |  |
| Setting | 5 | Describe the setting, locations, and relevant dates, including periods of recruitment, exposure, follow-up, and data collection | Patients under 24 months who were diagnosed with type 1 diabetes in the UK and a member of the British Diabetic Association (voluntary admittance) | Patients aged between 0 and 29, diagnosed with type 1 diabetes in Turin, Italy, and receiving treatment. | Cohort of IDDM patients diagnosed between 1965 and 1980, aged under 15, in Cuba. |  |  |
| Participants | 6 | (*a*) Give the eligibility criteria, and the sources and methods of selection of participants. Describe methods of follow-up | 302 patients; <2 yrs; UK; 1972 to 1981; Followed up 31-Dec-1989; Development of Diabetes pre 24 mths; British Diabetic Association Children’s Register; Contact with consultants; Family Health Services Authorities; GP | 1210 patients; 0-14 and 15-29; Turin, Italy; 1974 to 2000; Followed up 31-Dec-2003; Diagnosis of Type I Diabetes; Hospital Records of patient with Diabetes; Hospital Records; GPs; Demographic Files | 504 patients; under 15 yrs; Cuba; from 1965 to 1980; followed up 31-Dec-1991 through Population Registries; Diagnosis of Type I Diabetes; National Registry of IDDM; Diabetes Care Centre, National Institute of Endocrinology; |  |  |
|  |  | (*b*) For matched studies, give matching criteria and number of exposed and unexposed | Not Reported | Not Reported | Not Reported |  |  |
| Variables | 7 | Clearly define all outcomes, exposures, predictors, potential confounders, and effect modifiers. Give diagnostic criteria, if applicable | Age; Sex | Age; Sex | Age; Sex |  |  |
| Data sources/ measurement | 8* | For each variable of interest, give sources of data and details of methods of assessment (measurement). Describe comparability of assessment methods if there is more than one group | Death; demographic files; hospital discharges; autopsy records | Death; autopsy records; hospital discharges; | Death; autopsy records; hospital records |  |  |
| Bias | 9 | Describe any efforts to address potential sources of bias | Not reported | Ketoacidosis excluded in patient<30 yr, through death certificate analysis | Not reported |  |  |
| Study size | 10 | Explain how the study size was arrived at | From cohort size | From cohort size | Estimations through average number of incident cases |  |  |
| Quantitative variables | 11 | Explain how quantitative variables were handled in the analyses. If applicable, describe which groupings were chosen and why | Not reported | Not reported | Not reported |  |  |
| Statistical methods | 12 | (*a*) Describe all statistical methods, including those used to control for confounding | Not reported | SMR for all-cause mortality computed using italian pop as standard. Cox regression analysis | Kaplan-Meier method; Univariate Analysis; Cox-Mantel test; SMR |  |  |
|  |  | (*b*) Describe any methods used to examine subgroups and interactions | Not reported | Not reported | Not reported |  |  |
|  |  | (*c*) Explain how missing data were addressed | 8 Patients excluded for being too old | 3 patients excluded as lost to follow up | Not reported |  |  |
|  |  | (*d*) If applicable, explain how loss to follow-up was addressed | Not reported | Not reported | Not reported |  |  |
|  |  | (*e*) Describe any sensitivity analyses | Not reported | Not reported | Not reported |  |  |
| Results | | |  |  |  |  |  |
| Participants | 13* | (a) Report numbers of individuals at each stage of study—eg numbers potentially eligible, examined for eligibility, confirmed eligible, included in the study, completing follow-up, and analysed | 302 patients eligible and analysed | 1210 patients analysed | 504 patients analysed |  |  |
|  |  | (b) Give reasons for non-participation at each stage | Death | Death | Death |  |  |
|  |  | (c) Consider use of a flow diagram |  |  |  |  |  |
| Descriptive data | 14* | (a) Give characteristics of study participants (eg demographic, clinical, social) and information on exposures and potential confounders | Patients in the UK diagnosed with type 1 diabetes under 24 mths | Patients in Turin, Italy, aged between 0 and 29, diagnosed with diabetes and receiving care | Patients in Cuba, aged between 0-15 yrs, diagnosed with diabetes and receiving care |  |  |
|  |  | (b) Indicate number of participants with missing data for each variable of interest | 3 patients lost to follow up | Not reported | Not reported |  |  |
|  |  | (c) Summarise follow-up time (eg, average and total amount) | 8-17 years after onset | 30 years after onset | 25 years after onset |  |  |
| Outcome data | 15* | Report numbers of outcome events or summary measures over time | Mortality | Mortality | Mortality |  |  |
| Main results | 16 | (*a*) Give unadjusted estimates and, if applicable, confounder-adjusted estimates and their precision (eg, 95% confidence interval). Make clear which confounders were adjusted for and why they were included | SMR 5x cf. Gen pop. | Mean age of death was 30.2; mean diabetes duration was 13.4, in dead patient, and 13.3 in surviving. Overall SMR was 1.96. | No difference by gender or year of diagnosis, but sig. dif. In survival rate among age-at-diagnosis groups. 8.5x all-cause-mortality of gen. pop. Half deaths due to renal disease; Not possible to rule out confounders due to univariate analysis |  |  |
|  |  | (*b*) Report category boundaries when continuous variables were categorized | Not reported | Not reported | Not reported |  |  |
|  |  | (*c*) If relevant, consider translating estimates of relative risk into absolute risk for a meaningful time period | Not reported | Not reported | Not reported |  |  |
| Other analyses | 17 | Report other analyses done—eg analyses of subgroups and interactions, and sensitivity analyses | Not reported | Not reported | Not reported |  |  |
| Discussion | | |  |  |  |  |  |
| Key results | 18 | Summarise key results with reference to study objectives | See 16 | See 16 | See 16 |  |  |
| Limitations | 19 | Discuss limitations of the study, taking into account sources of potential bias or imprecision. Discuss both direction and magnitude of any potential bias | BDA Register not being complete, due to relying on voluntary contributions and being 20 years old. | Not reported | Impossibility of ruling out any contribution made to survival results by possible confounders because of use of univariate analysis. |  |  |
| Interpretation | 20 | Give a cautious overall interpretation of results considering objectives, limitations, multiplicity of analyses, results from similar studies, and other relevant evidence | Deaths since diagnosis were analysed in 310  children. Their SMR was approximately five times higher than in the general population. Given the small numbers of deaths in this cohort and the wide CI, our mortality results are similar to those reported in other studies comparing mortality in childhood diabetes to  that in the general population | This study shows that early mortality in an Italian incident population-based cohort of children and young adults with type 1 diabetes is two-fold higher than in the underlying population of similar age and sex. Most of the excess is due to potentially preventable causes of deaths such as ketoacidosis and hypoglycaemia. | A 71% cumulative survival rate at 25 years of IDDM duration; with no differences according to sex or calendar period of IDDM diagnosis. Cohort experienced 8.5 times the all-causes death rate, compared with the general population, with renal disease accounting for almost half the deaths. |  |  |
| Generalisability | 21 | Discuss the generalisability (external validity) of the study results | This cohort was established not only to investigate  mortality, but also natural history, particularly the occurrence  and severity of retinopathy and nephropathy  at present and prospectively. The results will provide  prognostic guidelines for paediatricians or physicians  caring for people with diabetes whose diagnosis was  made at a very young age. | Children and young adults with onset of type 1 diabetes in the period 1974-2000 experienced a two-fold higher mortality risk than Italian people of similar age and sex, particularly young adult-onset diabetic people. These data suggest that quality of care provided to young people in Italy with  type 1 diabetes should be improved. | Due to an early diagnosis; availability of insulin; qualified medical care, this study is consistent with previous findings from Israel where the cumulative mortality of young IDDM subjects resembles that of the general population during the first 15 years of disease duration. |  |  |
| Other information | | |  |  |  |  |  |
| Funding | 22 | Give the source of funding and the role of the funders for the present study and, if applicable, for the original study on which the present article is based | British Diabetic Association | AIRC and FIRMS | None cited |  |  |

|  | Item No | Recommendation | Feltbower et al, 2008 [27] | Florkowski et al, 2003 [12] | Gnavi et al, 2004 [28] |  |  |
| --- | --- | --- | --- | --- | --- | --- | --- |
| **Title and abstract** | 1 | (*a*) Indicate the study’s design with a commonly used term in the title or the abstract | Acute complications and drug misuse are important causes of death for children and young adults with type 1 diabetes: results from the Yorkshire Register of diabetes in children and young adults | Cause-specific and total mortality in the Canterbury (New Zealand) insulin-treated Diabetic Registry population: a 15-year follow-up study | Mortality and educational level among diabetic and non-diabetic population in the Turin Longitudinal Study: a 9-year follow-up |  |  |
|  |  | (*b*) Provide in the abstract an informative and balanced summary of what was done and what was found | OBJECTIVE: To examine mortality rates and causes of death among subjects diagnosed with type 1 diabetes aged <or=29 years. RESEARCH DESIGN AND METHODS: Subjects with type 1 diabetes from a population-based register in Yorkshire, U.K., diagnosed between 1978 and 2004 were linked to the U.K. National Health Service Central Register for death notifications. Deaths were coded using ICD-9 (1979-2000) and ICD-10 (2001-2005). Standardized mortality ratios (SMRs) were calculated using expected numbers of deaths from U.K. mortality rates by cause of death and age at diagnosis. RESULTS: A total of 4,246 individuals were followed up, providing 50,471 person-years of follow-up. Mean follow-up length was 12.8 years for individuals aged 0-14 years and 8.3 for those aged 15-29 years. Overall, 108 patients died, of whom 77 (71%) were male. A total of 74 (1.7/1,000 person-years) deaths occurred in inidividuals aged 0-14 years and 34 (4.6/1,000 person-years) in those aged 15-29 years. The SMR was 4.7 (95% CI 3.8-5.6) overall, similar for males and females, but higher for individuals aged 15-29 years (SMR 6.2 [95% CI 4.3-8.6]) compared with those aged 0-14 years (4.2 [3.3-5.3]). The SMR rose with increasing disease duration. A total of 47 of 108 deaths (44%) occurred from diabetes complications, 32 of which were acute and 15 chronic. Twenty-two percent (n = 24) of deaths were attributed to accidents or violence (SMR 2.1 [95% CI 1.4-3.2]), including six suicides. Sixteen percent of all deaths were related to drug misuse (including insulin but excluding tobacco and alcohol) (SMR 6.4 [95% CI 3.7-10.2]). CONCLUSIONS: Subjects with type 1 diabetes diagnosed under 30 years of age had a 4.7-fold excess mortality risk. Nearly half of the deaths were due to acute or chronic complications of diabetes. Drug misuse-related deaths may be an emerging trend in this population warranting further investigation. | Aims To establish all-cause and cause-specific death rates, and risk factors for mortality in insulin-treated diabetic individuals living in the province of Canterbury, New Zealand. Methods Insulin-treated diabetic subjects (n=995) on the Canterbury Diabetes Registry were followed up over 15 years and vital status determined. Death rates were standardized and hazard regression was used to model the effects of demographic covariates on relative survival time. Results There were 419 deaths in 11 226.3 person-years of follow-up with a standardized mortality ratio (SMR) of 2.0 (95% confidence interval (CI) 1.8-2.2). Relative mortality was greatest for the group aged 0-29 years (SMR 3.0 (95% CI 2.4-3.7)). After controlling for diabetes duration and gender, a 10-year increment in age of onset was associated with a 33% decrease in relative hazard (95% CI 29-36%), indicating that excess mortality due to diabetes declines with rising age of onset. After controlling for age of onset and gender, each 10-year increment in duration of diabetes is associated with a 26% decrease in relative hazard (95% CI 24-29%), indicating that with longer survival the mortality hazard approaches the general population hazard. Relative mortalities were increased for cardiovascular, renal and respiratory disease, but not malignancy. Relative mortality from acute metabolic complications was increased in the subgroup with age of onset of diabetes <30 years and requiring insulin within 1 year of diagnosis. Conclusions Mortality rates are high for insulin-treated diabetic individuals relative to the general population. | BACKGROUND: People in a lower social position have a higher prevalence of unhealthy behaviour, more difficult access to healthcare, and lower compliance with drug treatment; as a consequence, social differences in mortality are likely to be higher in people with diabetes compared with the non-diabetic population. We compared diabetics with non-diabetics in terms of mortality and social differences in mortality. METHODS: In all, 31 264 residents in Turin (northern Italy), who were > or =20 years old, registered in the local diabetes register between 1991 and 1999. They were followed up from recruitment to December 1999, and their cause-specific mortality by educational level was analysed. This was compared with that of the local non-diabetic population. Diabetes was classified as type 1 (< or =35 years at diagnosis) or type 2 (>35 years). RESULTS: For type 1 diabetes, the all-cause standardized mortality ratio (SMR) was 197.6 (95% CI:155.7, 247.4) in men and 336.0 (95% CI:259.3, 428.2) in women; for type 2 diabetes, the all-cause SMR was 142.8 (95% CI:138, 147.6) in men and 143.4 (95% CI:138.5, 148.5) in women. Whereas social differences in mortality were evident among non-diabetic men and women for all causes of death considered, no significant differences were found among diabetic women. Mortality was slightly increased among less educated diabetic men, particularly for neoplasms, although this gradient was less steep than that among non-diabetics. CONCLUSIONS: These results suggest that the regular clinical follow-up and health education provided by the local network of diabetic centres might play an important role in confronting the adverse effects of diabetes and in reducing social differences in health. |  |  |
| Introduction | | |  |  |  |  |  |
| Background/rationale | 2 | Explain the scientific background and rationale for the investigation being reported | The literature contains a number of  studies focusing on mortality among  those who develop type 1 diabetes in  childhood (4 –15), but few data exist on  risk and causes of death in those diagnosed  before 30 years of age. One U.K.  analysis of individuals aged 0–29 years  (7) demonstrated an excess mortality risk  ranging from 2 to 6 times that of the general  population for individuals aged 40  years. Older teenagers and young adults  constitute a transitional group between  children and adults and often fall out of  health care provision. They have also  been neglected in research studies and,  due to differences in the delivery of care  among individuals aged 15–29 years,  mortality risks may differ from those in  children aged 0–14 years. | Although there have been improvements in the prognosis of people with diabetes [1Ð6], mortality rates for people with diabetes are on average two to four-fold higher than those for the general population [7Ð9]. Life expectancy for individuals, both male and female, with Type 1 diabetes is reduced by approximately one-third [5,10]. Premature mortality is most often attributed to the long-term renal and cardiovascular complications of the disorder [1,7,9]. | In most countries, including Italy, social position has been shown to be inversely related to certain types of unhealthy behaviour, and to access to high quality care; thus social inequalities in mortalitycan be expected to be greater among people with diabetes, compared with those without diabetes. |  |  |
| Objectives | 3 | State specific objectives, including any pre-specified hypotheses | The aim of the study was to obtain  information on the causes of death in  young people diagnosed under 30 years  of age, thereby informing clinicians about  how better to manage their patients  prospectively. | The primary aim was to establish the overall, cause-specific and age-sex specific death rates of insulin using diabetic individuals living in Canterbury after 15 years of follow-up. The next aim was to compare these death rates with those of the general population and to examine the relative importance of basic demographic and clinical epidemiological risk factors such as gender, attained age, age at diagnosis, duration of diabetes as significant predictors of mortality. Finally, to determine if differences in mortality exist between three designated clinical subgroups, namely juvenile onset Type 1 diabetes, adult-onset insulin-dependent diabetes, and those with delayed requirement for insulin treatment. | To compare diabetics with non-diabetics in terms of mortality and social differences in mortality, we conducted a study among people with diabetes living in the city of Turin, Italy, and compared them with Turin residents without diabetes. |  |  |
| Methods | | |  |  |  |  |  |
| Study design | 4 | Present key elements of study design early in the paper | Diabetic patient register identified patients diagnosed pre-15 yrs, and patients under 30 with diabetes; | Division of patients into age groups, examining attained age, age of onset and diabetes duration. | Division into type 1 and type 2 diabetic patients; >20 yrs old in Turin, Italy, comparing mortality by educational level; against the general population |  |  |
| Setting | 5 | Describe the setting, locations, and relevant dates, including periods of recruitment, exposure, follow-up, and data collection | Yorkshire, UK; from 1978 to 2004; follow up using hospital records, local registries; followed up on 31-Dec-05 through the NHSCR | Patients from Canterbury, NZ, as of 1-Jan-1984; followed up in 1-Jan-1999 | Patients from Turin, Italy, as of 1991-1999, with a follow up in 31-Dec-1999. |  |  |
| Participants | 6 | (*a*) Give the eligibility criteria, and the sources and methods of selection of participants. Describe methods of follow-up | 4256 patients; between 0-29 yrs; UK; from 1978 to 2004; Diagnosis of diabetes; on the UK National Health Service Central Register; followed up on 31-Dec-05 through the NHSCR | 995 patients; all ages; Canterbury, NZ; if they resided locally as of 1-jan-1984; long-term insulin treatment; Followed up on 1-Jan-1999 via National Hospital records; Canterbury diabetes registry | 31264 patients; >20 yrs; Turin, Italy; from 1991-1999; followed up on 31-Dec-1999 with Turin Mortality Register; Registered with Piemonte Diabetes Register; sourced through Turin Longitudinal Study |  |  |
|  |  | (*b*) For matched studies, give matching criteria and number of exposed and unexposed | Not reported | Not reported | Not reported |  |  |
| Variables | 7 | Clearly define all outcomes, exposures, predictors, potential confounders, and effect modifiers. Give diagnostic criteria, if applicable | Age; Sex | Age; Sex | Age; Sex |  |  |
| Data sources/ measurement | 8* | For each variable of interest, give sources of data and details of methods of assessment (measurement). Describe comparability of assessment methods if there is more than one group | NHSCR | National Hospital records; canterbury diabetes registry | Turin Mortality Register |  |  |
| Bias | 9 | Describe any efforts to address potential sources of bias | Not reported | Not reported | Not reported |  |  |
| Study size | 10 | Explain how the study size was arrived at | From cohort size | From cohort size | From cohort size |  |  |
| Quantitative variables | 11 | Explain how quantitative variables were handled in the analyses. If applicable, describe which groupings were chosen and why | Not reported | Not reported | Not reported |  |  |
| Statistical methods | 12 | (*a*) Describe all statistical methods, including those used to control for confounding | SMRs; CI's derived assuming a Poisson distribution | SMRs; Poisson distribution; hazard regression models; | SMR; survival analysis using Cox proportional hazards models; Confounded by Area of birth |  |  |
|  |  | (*b*) Describe any methods used to examine subgroups and interactions | Not reported | Not reported | Not reported |  |  |
|  |  | (*c*) Explain how missing data were addressed | Not reported | 99 patient lost to follow up were excluded | Not reported |  |  |
|  |  | (*d*) If applicable, explain how loss to follow-up was addressed | Not reported | Not reported | Not reported |  |  |
|  |  | (*e*) Describe any sensitivity analyses | Not reported | Not reported | Not reported |  |  |
| Results | | |  |  |  |  |  |
| Participants | 13* | (a) Report numbers of individuals at each stage of study—eg numbers potentially eligible, examined for eligibility, confirmed eligible, included in the study, completing follow-up, and analysed | 4246 patients analysed | 995 patients analysed | 31264 patients analysed |  |  |
|  |  | (b) Give reasons for non-participation at each stage | Mortality | Mortality | Mortality |  |  |
|  |  | (c) Consider use of a flow diagram |  |  |  |  |  |
| Descriptive data | 14* | (a) Give characteristics of study participants (eg demographic, clinical, social) and information on exposures and potential confounders | Patients <30 yrs; residing in Yorkshire, with Type 1 diabetes | Any age patients; Residing in Canterbury, NZ; with Type 1 Diabetes | Patients >20yrs; residing in Turin, Italy; with type 1 diabetes |  |  |
|  |  | (b) Indicate number of participants with missing data for each variable of interest | Not reported | Not reported | Not reported |  |  |
|  |  | (c) Summarise follow-up time (eg, average and total amount) | 27 years since onset | 15 years since onset | 10 years since onset |  |  |
| Outcome data | 15* | Report numbers of outcome events or summary measures over time | 15 outcomes | 18 outcomes | 18 outcomes |  |  |
| Main results | 16 | (*a*) Give unadjusted estimates and, if applicable, confounder-adjusted estimates and their precision (eg, 95% confidence interval). Make clear which confounders were adjusted for and why they were included | 108 patient died; 71% male; SMR 4.2 for 0-14 cf. 6.2 for 15-29. 44% deaths from complications; 6 suicides | 419 patient died; SMR female 2.1 cf. SMR male 1.9 | 6814 patient died; Type I Diabetes risk of death double cf. gen. pop. Tripled in women. Education level inverse to mortality rate |  |  |
|  |  | (*b*) Report category boundaries when continuous variables were categorized | Not Reported | Not Reported | Not Reported |  |  |
|  |  | (*c*) If relevant, consider translating estimates of relative risk into absolute risk for a meaningful time period | Not Reported | Not Reported | Not Reported |  |  |
| Other analyses | 17 | Report other analyses done—eg analyses of subgroups and interactions, and sensitivity analyses | Not Reported | Not Reported | Not Reported |  |  |
| Discussion | | |  |  |  |  |  |
| Key results | 18 | Summarise key results with reference to study objectives | See 16 | See 16 | See 16 |  |  |
| Limitations | 19 | Discuss limitations of the study, taking into account sources of potential bias or imprecision. Discuss both direction and magnitude of any potential bias | Cause of death uncertain in 19 cases, and this is a significant number of cases. A proportion of the fatalities attributed to uncertain causes are likely to have been related to diabetes and accidents/violence, suggesting that we may have underestimated the true number in our diabetes population. | Not reported | Specifically, the PDR could be incomplete, which would reduce the social gradient if people with a lower social position with less severe diabetes or people with a higher social position with more severe diabetes were preferentially included. For type 2 diabetes, it is likely that both of these conditions were present. People with a higher social position with mild diabetes could be less interested than the more disadvantaged in the economic advantages of registering with the PDR, making them less likely to register, which would lead to an overestimation of mortality among diabetics with a high social position. For type 1 diabetes, this source of bias is probably less relevant, given the higher severity of the disease, which involves more frequent clinical or biochemical evaluation. On the other hand, only 936 (1.2%) of deaths in the non-diabetic population were due to diabetes, which can be reasonably attributed, at least in part, to previously undiagnosed diabetes being discovered at the moment of death. Moreover, if the selection bias were strong, there would be no evident social gradient in mortality due to diabetes in the non-diabetic population, yet in our study (Table 3) a social gradient was present and it showed the same direction as that in the diabetic population. Secondly, we used a rather crude definition for type 1 and type 2 diabetes  (i.e. based on age of diagnosis).1 |  |  |
| Interpretation | 20 | Give a cautious overall interpretation of results considering objectives, limitations, multiplicity of analyses, results from similar studies, and other relevant evidence | Risk of death for children and young adults with type 1 diabetes in Yorkshire was four and six times that of the background population. Acute complications accounted for approximately one-third of all deaths, emphasizing that, despite modern intensive education and insulin treatment, such deaths continue to occur.  Similarly, chronic complications including cardiac, renal, and cerebrovascular causes continued to arise and subsequently proved fatal. | Our data showed that excess mortality due to diabetes declines with age of onset. Our data also showed that after controlling for age of onset and gender, each 10-year increment in duration of diabetes is associated with a 26% decrease in relative hazard. Thus, the longer a diabetic patient survives the more their mortality hazard approaches that of the general population. | With regard to differences in mortality, we confirmed that people with diabetes have an increased risk of death, specifically, a 42% increase, both in men and in women, for type 2 diabetes, and a 98% increase in men and a 236% increase in women for type 1 diabetes. People with a higher educational level had a lower mortality. |  |  |
| Generalisability | 21 | Discuss the generalisability (external validity) of the study results | Identified a propensity for young-adult subjects to misuse drugs or take insulin overdoses. A previous survey of young adults with type 1 diabetes found that street drug misuse was common (28). | All-cause mortality in Canterbury’s insulin-treated diabetic population after 15 years of follow-up was found to be double that of the general population, standardizing for age and gender differences. This level of relative mortality risk is similar to that recorded during the 1980s in Tasmania (SMR of 2.2, 95% CIs 2.0Ð2.4) after 8.5 years of follow-up [9], a population with similar demographic and clinical characteristics to that in Canterbury. | Our finding of a less-marked increase in the risk of mortality among people with diabetes compared with the increases reported in previously published studies,1,2 together with the absence of a significant social gradient, needs to be explained. In Italy, although the National Health Service ensures that all citizens are provided with quality healthcare at nearly no cost, there do exist social differences in health.21 |  |  |
| Other information | | |  |  |  |  |  |
| Funding | 22 | Give the source of funding and the role of the funders for the present study and, if applicable, for the original study on which the present article is based | UK department of health | Lotteries Distribution Board | Italian Ministry of Health |  |  |

|  | Item No | Recommendation | Grausland et al, 2010 [29] | Harjutsalo et al, 2011 [30] | Joner & Patrick, 1991 [17] |  |  |
| --- | --- | --- | --- | --- | --- | --- | --- |
| **Title and abstract** | 1 | (*a*) Indicate the study’s design with a commonly used term in the title or the abstract | Risk factors for mortality and ischemic heart disease in patients with long-term type 1 diabetes | Time trends in mortality in patients with type 1 diabetes: nationwide population based cohort study | The mortality of children with type 1 (insulin-dependent) diabetes mellitus in Norway, 1973-1988 |  |  |
|  |  | (*b*) Provide in the abstract an informative and balanced summary of what was done and what was found | AIMS: The purpose of this study is to evaluate the effect of glycemic regulation, dyslipidemia, and renal dysfunction on mortality (all-cause and cardiovascular) and ischemic heart disease (IHD) in a long-term follow-up of a population-based cohort of Danish type 1 diabetic patients with at least 20 years of diabetes. METHODS: A population-based cohort of type 1 diabetic patients was identified as of July 1, 1973 (n=727). In 1993 to 1996, the cohort was reassessed and baseline data were collected from blood and urine samples in 389 patients. Mean (glycemic regulation and lipids) and highest values (creatinine and albuminuria) of the baseline period were used to predict mortality and IHD between baseline and 2006. Data of mortality and morbidity were provided by the Danish Civil Registration System, the Danish Causes of Death Registry, and the Danish National Patient Registry. RESULTS: At the follow-up in 2006, 256 patients (65.8%) were still alive. In a statistical model adjusted for age, sex and duration of diabetes, the following parameters were related to all-cause mortality and cardiovascular mortality: glycemic regulation, triglycerides, low-density lipoprotein cholesterol, high-density lipoprotein cholesterol (inversely), total cholesterol, creatinine, and macroalbuminuria. Furthermore, all markers except macroalbuminuria were associated with IHD. Microalbuminuria at baseline was not related to any of the endpoints. CONCLUSIONS: Glycemic regulation, dyslipidemia, and renal dysfunction were all related to mortality and IHD in a 13-year follow-up of long-term Danish type 1 diabetic patients. These results underscore the better outcome for tightly regulated type 1 diabetic patients, even in long-term survivors. Copyright 2010 Elsevier Inc. All rights reserved. | OBJECTIVE: To examine short and long term time trends in mortality among patients with early onset (age 0-14 years) and late onset (15-29 years) type 1 diabetes and causes of deaths over time. DESIGN: Population based nationwide cohort study. SETTING: Finland. PARTICIPANTS: All Finnish patients diagnosed as having type 1 diabetes below age 30 years between 1970 and 1999 (n = 17,306). MAIN OUTCOME MEASURES: Crude mortality, standardised mortality ratios, time trends, and cumulative mortality. RESULTS: A total of 1338 deaths occurred during 370,733 person years of follow-up, giving an all cause mortality rate of 361/100,000 person years. The standardised mortality ratio was 3.6 in the early onset cohort and 2.8 in the late onset cohort. Women had higher standardised mortality ratios than did men in both cohorts (5.5 v 3.0 in the early onset cohort; 3.6 v 2.6 in the late onset cohort). The standardised mortality ratio at 20 years' duration of diabetes in the early onset cohort decreased from 3.5 in the patients diagnosed in 1970-4 to 1.9 in those diagnosed in 1985-9. In contrast, the standardised mortality ratio in the late onset cohort increased from 1.4 in those diagnosed in 1970-4 to 2.9 in those diagnosed in 1985-9. Mortality due to chronic complications of diabetes decreased with time in the early onset cohort but not in the late onset cohort. Mortality due to alcohol related and drug related causes increased in the late onset cohort and accounted for 39% of the deaths during the first 20 years of diabetes. Accordingly, mortality due to acute diabetic complications increased significantly in the late onset cohort. CONCLUSION: Survival of people with early onset type 1 diabetes has improved over time, whereas survival of people with late onset type 1 diabetes has deteriorated since the 1980s. Alcohol has become an important cause of death in patients with type 1 diabetes, and the proportion of deaths caused by acute complications of diabetes has increased in patients with late onset type 1 diabetes. | The mortality status of all individuals in Norway with the onset of Type 1 (insulin-dependent) diabetes mellitus from 1973 through 1982 and age at onset below 15 years was determined as of 1 July 1988. Of the 1908 cases included in the follow-up, 20 had died (15 males and 5 females) and 10 had emigrated. A two-fold increased risk for early mortality was exhibited among this cohort. Life-table analyses did not find sex or age at onset of Type 1 diabetes to be statistically significant predictors of survival when controlling for diabetes duration. A review of death certificates revealed that accidents and suicides accounted for 40% of the deaths in the total cohort and that this cause of death occurred only among male subjects. Acute diabetes related complications were the underlying causes of death for 35% of the subjects. Diabetic renal disease and death by cardiovascular disease were not documented in this young cohort with a maximum age of 30 years and maximum diabetes duration of 15.5 years. This is the first mortality report of a population-based registered cohort of Type 1 diabetic patients for Norway. While still being at increased risk for premature death, this cohort appears to be at decreased risk of early death when compared to a cohort of young diabetic patients from Oslo, Norway diagnosed in 1925-1955, suggesting improvements in the survival of individuals with Type 1 diabetes in Norway. |  |  |
| Introduction | | |  |  |  |  |  |
| Background/rationale | 2 | Explain the scientific background and rationale for the investigation being reported | The prevalence of diabetes in Denmark has increased from 114 000 in 1997 to 227 000 in  2006 and the number is expected to rise to 470 000–625 000 by 2025 (Green 2008). Of these, approximately 10% are estimated to be type 1 diabetic patients. | The age at onset of diabetes may influence the risk of late complications of diabetes. In particular, patients with onset of diabetes after age 15 have been observed to have a lower risk of diabetic nephropathy and end stage renal disease than do patients diagnosed during adolescence.5-7 Better long term survival could thus be expected in people diagnosed as having diabetes after adolescence. | In spite of dramatic increases in the life expectancy of children diagnosed with Type 1 (insulin-dependent) diabetes mellitus since the introduction of insulin therapy [1], studies continue to confirm an increased mortality among subjects with Type i diabetes compared to healthy subjects. This increased risk for death at a given age appears to be of a seven-fold magnitude in the United States |  |  |
| Objectives | 3 | State specific objectives, including any prespecified hypotheses | Evaluate the all-cause mortality rates and the influence of sex, duration of diabetes and calendar year of diagnosis in a 33-year follow-up | The aim of this study was therefore to investigate short term and long term time trends in mortality in patients diagnosed as  having early onset (age 0-14 years) and late onset (15-29 years) type 1 diabetes in Finland, the country with the world’s highest incidence of type 1 diabetes. | The present study is the first to investigate the  mortality of young diabetic patients in Norway and is based upon a nationwide population-based registry covering the time period from 1973 through 1982. |  |  |
| Methods | | |  |  |  |  |  |
| Study design | 4 | Present key elements of study design early in the paper | Mortality was examined in the entire cohort of 727 type 1 diabetic patients in 1973–2006. All patients were followed from 1 July 1973 to the date of death, loss to follow-up (due to emigration from Denmark or refusal to provide data for scientific studies) or censoring at 6 November 2006, whichever came first. | We identified 17 306 people diagnosed as having type 1 diabetes below 30 years of age between 1970 and 1999 from the drug  reimbursement register. | All new-onset cases of Type I diabetes occurring in Norwegian children age 0-14 years during the time period 1 January 1973 to  31 December 1982 were registered retrospectively |  |  |
| Setting | 5 | Describe the setting, locations, and relevant dates, including periods of recruitment, exposure, follow-up, and data collection | See 6 | See 6 | See 6 |  |  |
| Participants | 6 | (*a*) Give the eligibility criteria, and the sources and methods of selection of participants. Describe methods of follow-up | 727 patients; diabetes diagnosed <30 yrs; in Denmark; 1973-2006; followed up on 6-nov-2006 via Danish Civil Registration System; Diagnosis of Type I Diabetes; Fyn County Denmark; pre 30 yr; Selected via Danish Civil Registration System; | 17306 patients; Finland; from 1970-1999; Diagnosis of Diabetes; permanent entitlement for insulin; under 30 yrs; Hospital Discharge Register; National Institute for Health and Welfare; followed up on 2007 via Finish Cause of Death Register | 1908 patients under 14; Norway; between 1973-1982; with a diagnosis of diabetes; selected through Hospital data; National Insurance Institution; followed up 1-jul-1988 through Mortality Registers; Hospital Records; |  |  |
|  |  | (*b*) For matched studies, give matching criteria and number of exposed and unexposed | Not reported | Not reported | Not reported |  |  |
| Variables | 7 | Clearly define all outcomes, exposures, predictors, potential confounders, and effect modifiers. Give diagnostic criteria, if applicable | Age; Sex | Age; Sex | Age; Sex |  |  |
| Data sources/ measurement | 8* | For each variable of interest, give sources of data and details of methods of assessment (measurement). Describe comparability of assessment methods if there is more than one group | Danish Civil Registration System | Finish Cause of Death Register | Mortality Registers; Hospital records |  |  |
| Bias | 9 | Describe any efforts to address potential sources of bias | Not reported | Exclusion of cases in hospital discharge register with a code indicating possible secondary diabetes before diagnosis of diabetes | Exclusion of cases where diagnosis date uncertain; diabetes developed as a secondary cause; chronic conditions |  |  |
| Study size | 10 | Explain how the study size was arrived at | From cohort size | From cohort size | From cohort size |  |  |
| Quantitative variables | 11 | Explain how quantitative variables were handled in the analyses. If applicable, describe which groupings were chosen and why | Not reported | Not reported | Not reported |  |  |
| Statistical methods | 12 | (*a*) Describe all statistical methods, including those used to control for confounding | SMR; Patients were censored at time of first registered ischaemic cardiac event | Poisson Regression Modelling; SMR; Kaplan-Meier; | Cox proportional hazards regression model; SMR; |  |  |
|  |  | (*b*) Describe any methods used to examine subgroups and interactions | Not reported | Not reported | Not reported |  |  |
|  |  | (*c*) Explain how missing data were addressed | Not reported | Not reported | Not reported |  |  |
|  |  | (*d*) If applicable, explain how loss to follow-up was addressed | Patients removed from cohort | Not reported | Not reported |  |  |
|  |  | (*e*) Describe any sensitivity analyses | Not reported | Not reported | Not reported |  |  |
| Results | | |  |  |  |  |  |
| Participants | 13* | (a) Report numbers of individuals at each stage of study—eg numbers potentially eligible, examined for eligibility, confirmed eligible, included in the study, completing follow-up, and analysed | 727 patients initial; 312 patients survived until follow up period | 17306 patients initial; 1338 deaths pre follow up period | 1908 patients initial; 20 deaths |  |  |
|  |  | (b) Give reasons for non-participation at each stage | Death | Death | Death |  |  |
|  |  | (c) Consider use of a flow diagram |  |  |  |  |  |
| Descriptive data | 14* | (a) Give characteristics of study participants (eg demographic, clinical, social) and information on exposures and potential confounders | See 6 | See 6 | See 6 |  |  |
|  |  | (b) Indicate number of participants with missing data for each variable of interest | Not reported | Not reported | Not reported |  |  |
|  |  | (c) Summarise follow-up time (eg, average and total amount) | 33 years | 30 years | 15 years |  |  |
| Outcome data | 15* | Report numbers of outcome events or summary measures over time | 18 outcomes | 24 outcomes | 18 outcomes |  |  |
| Main results | 16 | (*a*) Give unadjusted estimates and, if applicable, confounder-adjusted estimates and their precision (eg, 95% confidence interval). Make clear which confounders were adjusted for and why they were included | 386 patient died; MR 22.3/1000 person years; | 1338 patient died; SMR 3.6 in early onset cf. 2.8 late onset; 39% of deaths due to alcohol and drug complications during the first 20 years of diabetes | 2x increase in early mortality risk; accidents and suicides responsible for 40% of deaths; diabetes-related complications account for 35% of deaths |  |  |
|  |  | (*b*) Report category boundaries when continuous variables were categorized | Not reported | Not reported | Not reported |  |  |
|  |  | (*c*) If relevant, consider translating estimates of relative risk into absolute risk for a meaningful time period | Not reported | Not reported | Not reported |  |  |
| Other analyses | 17 | Report other analyses done—eg analyses of subgroups and interactions, and sensitivity analyses | Not reported | Not reported | Not reported |  |  |
| Discussion | | |  |  |  |  |  |
| Key results | 18 | Summarise key results with reference to study objectives | One of the main findings of the present study was a 3.4-fold increased mortality in Danish type 1 diabetic patients as compared to the general population. | A total of 1338 deaths occurred during 370 733 person years of follow-up, giving an all-cause mortality rate of 361 (95% confidence interval 342 to 382) per 100 000 person years. | 2x increase in early mortality risk; accidents and suicides responsible for 40% of deaths; diabetes-related complications account for 35% of deaths |  |  |
| Limitations | 19 | Discuss limitations of the study, taking into account sources of potential bias or imprecision. Discuss both direction and magnitude of any potential bias | However, data reporting the causes of death in our study should be read with caution. These data are in general based on death-reports made by hospital doctors who are often without thorough knowledge of the patients. This may cause misclassification.  Likewise, even for doctors who know the patients well, the definition of specific causes of death, i.e. heart disease, may cause difficulties per se. | Lack of precise information about diabetes type; | Not reported |  |  |
| Interpretation | 20 | Give a cautious overall interpretation of results considering objectives, limitations, multiplicity of analyses, results from similar studies, and other relevant evidence | In this study, diabetes was mentioned as the underlying cause of death for most of the patients who died in 1973–2006. Heart diseases were only given as the cause of death for one in five patients. | This study provides a contemporary picture of mortality in early onset as well as late onset type 1 diabetes between 1970 and  2007 in Finland, the country with the highest incidence of type 1 diabetes in the world.8 9 An encouraging finding is that survival in the early onset cohort has improved in those patients most recently diagnosed as having diabetes. This was mainly because of a decrease in mortality due to chronic complications of diabetes. This encouraging finding in the early onset cohort is, however, overshadowed by the unfavourable findings in the late onset cohort, in which we saw an increasing trend in both short term and long term standardised mortality ratios. | There are several potential reasons for this apparent considerable decrease in early mortality among patients with Type i diabetes in Norway. Factors such as improved  physician and patient education regarding diabetes, easy access to free, high quality health care, and technological advances in glucose monitoring, diabetes care and treatment of diabetes complications are likely to have all played a role in the lengthened survival of individuals with Type i diabetes in Norway. |  |  |
| Generalisability | 21 | Discuss the generalisability (external validity) of the study results | Similar data has been reported from other European countries such as the United Kingdom (Laing et al. 1999; Soedamah-Muthu et al. 2006), Finland (Asao et al. 2003), Israel (Laron-Kenet et al. 2001) and Norway (Skrivarhaug et al. 2006). Data from USA (Moss et al. 1991) and Japan (Asao et al. 2003) showed even higher MR of 7.5 and 12.9, respectively. | Compared to other Finnish studies, increased survival only occurred in patient with early onset age | A study on the mortality of diabetic patients diagnosed between 1925-1955 from Oslo, Norway and followed until anniversary of discharge in 1961 was published by Westlund in monograph form in 1969 [14]. Although  the methods employed are not directly comparable to the present study, it is the only previous study that presents mortality data for diabetic subjects for Norway. The mortality  rate for young diabetic subjects ( < 30 years old at follow-up) was approximately 1000/100,000 person-years (calculated by the authors). Comparing this to the rate found in the present study (108/100,000 person-years), there appears to be a major reduction in the mortality associated with Type i diabetes in Norway. |  |  |
| Other information | | |  |  |  |  |  |
| Funding | 22 | Give the source of funding and the role of the funders for the present study and, if applicable, for the original study on which the present article is based | Not reported | Grants from Folkhalsan Research Fund, Wilhelm and Else Stockmann Foundation, and Liv och Halsa Foundation. | National Institutes of Health Grant; The Norwegian Research Council for Science and Humanities grant; Norwegian Diabetes Association Grant |  |  |

|  | Item No | Recommendation | Laing et al, 1999 [31] | McNally et al, 1995 [14] | Morrish et al, 2001 [21] |  |  |
| --- | --- | --- | --- | --- | --- | --- | --- |
| **Title and abstract** | 1 | (*a*) Indicate the study’s design with a commonly used term in the title or the abstract | The British Diabetic Association Cohort Study, I: all-cause mortality in patients with insulin-treated diabetes mellitus. | Trends in mortality of childhood-onset insulin-dependent diabetes mellitus in Leicestershire: 1940-1991 | Mortality and causes of death in the WHO Multinational Study of Vascular Disease in Diabetes |  |  |
|  |  | (*b*) Provide in the abstract an informative and balanced summary of what was done and what was found | AIMS: To assess mortality in patients with diabetes incident under the age of 30 years.  METHODS: A cohort of 23 752 diabetic patients diagnosed under the age of 30 years from throughout the United Kingdom was identified during 1972-93 and followed up to February 1997. Following notification of deaths during this period, age- and sex-specific mortality rates, attributable risks and standardized mortality rates were calculated.  RESULTS: The 23 752 patients contributed a total of 317 522 person-years of follow-up, an average of 13.4 years per subject. During follow-up 949 deaths occurred in patients between the ages of 1 and 84 years, 566 in males and 383 in females. All-cause mortality rates in the patients with diabetes exceeded those in the general population at all ages and within the cohort were higher for males than females at all ages except between 5 and 15 years. The relative risk of death (standardized mortality ratio, SMR), was higher for females than males at all ages, being 4.0 (95% CI 3.6-4.4) for females and 2.7 (2.5-2.9) for males overall, but reaching a peak of 5.7 (4.7-7.0) in females aged 20-29, and of 4.0 (3.1-5.0) in males aged 40-49. Attributable risks, or the excess deaths in persons with diabetes compared with the general population, increased with age in both sexes.  CONCLUSIONS: This is the first study from the UK of young patients diagnosed with diabetes that is large enough to calculate detailed age-specific mortality rates. This study provides a baseline for further studies of mortality and change in mortality within the United Kingdom. | The relative risk of death by calendar date of diagnosis was investigated in a population-based incident cohort of 845 (463 males:382 females) IDDM diagnosed in Leicestershire before the age of 17 years between 1940 and 1989. The mortality status of 844 (99.9%) patients was determined as of the 31 December 1991, representing 14,346 person-years of risk. Trends in relative risk of death were investigated using Cox proportional hazards modelling for within cohort comparisons and age/sex and calendar time adjusted standardized mortality ratios (SMR) using generalized linear modelling for external comparisons. Median age at diagnosis was 10 years (range 3 months to 16 years); median duration of diabetes 15 years (range 1-51 years). Forty-four patients had died (5.2%; median age at death 31 years, range 11-51 years). A further four patients died at presentation (within 24 h) from ketoacidosis and are excluded from all analyses. Calendar date of diagnosis was found to be an important predictor of mortality. Adjusting for attained age there was evidence of a decline in relative risk of death with calendar date of diagnosis of 3.4% (95% CI, 0.005-6.9%) per annum, equivalent to a 32% fall per decade (95% CI, 5-51%), or 84% (95% CI, 21-97) from 1940 to 1989. The data are consistent with a large fall in mortality between the 1940s and 1950s representing over 50% of the total reduction in mortality between 1940 and 1991. Neither sex nor age at diagnosis were significant predictors of mortality. Over the study period 1940-89 the SMR (male and female combined) fell from 981 (541-1556) to 238 (60-953) relative to the general population. This population-based study shows that the prognosis for Type 1 (insulin-dependent) diabetes mellitus has improved markedly over the period 1940-1991. | AIMS/HYPOTHESIS: We aimed to examine the mortality rates, excess mortality and causes of death in diabetic patients from ten centres throughout the world. METHODS: A mortality follow-up of 4713 WHO Multinational Study of Vascular Disease in Diabetes (WHO MSVDD) participants from ten centres was carried out, causes of death were ascertained and age-adjusted mortality rates were calculated by centre, sex and type of diabetes. Excess mortality, compared with the background population, was assessed in terms of standardised mortality ratios (SMRs) for each of the 10 cohorts. RESULTS: Cardiovascular disease was the most common underlying cause of death, accounting for 44 % of deaths in Type I (insulin-dependent) diabetes mellitus and 52 % of deaths in Type II (non-insulin-dependent) diabetes mellitus. Renal disease accounted for 21% of deaths in Type I diabetes and 11% in Type II diabetes. For Type I diabetes, all-cause mortality rates were highest in Berlin men and Warsaw women, and lowest in London men and Zagreb women. For Type II diabetes, rates were highest in Warsaw men and Oklahoma women and lowest in Tokyo men and women. Age adjusted mortality rates and SMRs were generally higher in patients with Type I diabetes compared with those with Type II diabetes. Men and women in the Tokyo cohort had a very low excess mortality when compared with the background population. CONCLUSION/INTERPRETATION: This study confirms the importance of cardiovascular disease as the major cause of death in people with both types of diabetes. The low excess mortality in the Japanese cohort could have implications for the possible reduction of the burden of mortality associated with diabetes in other parts of the world. |  |  |
| Introduction | | |  |  |  |  |  |
| Background/rationale | 2 | Explain the scientific background and rationale for the investigation being reported | If diabetic mortality, and changes in diabetic mortality, are to be studied accurately in the United Kingdom, then baseline mortality rates specific to the UK population must be  established. To date, there has not been a cohort study of young patients with diabetes with sufficient numbers to examine mortality within narrow age strata. | Despite early improvements in short-term  survival significant numbers were dying prematurely from renal failure and vascular diseases by the 1940’s published studies dealing with mortality in insulin-dependent  (Type 1) diabetes mellitus (IDDM) relate to patients diagnosed soon after the introduction of insulin, or to patients receiving treatment in the US. | Difficulty in gaining information about deaths in diabetic patients, as on death certificates, diabetes is often omitted from the certificate. |  |  |
| Objectives | 3 | State specific objectives, including any prespecified hypotheses | This paper describes the foundation of a cohort of over 23 000 patients with insulin-treated diabetes, set up under the auspices of the British Diabetic Association (BDA), and  reports all-cause mortality results within 10 year age strata. | There are no comparative studies of mortality for childhood-onset IDDM in the United Kingdom, so the present study has investigated trends in mortality of childhood-onset IDDM in the United Kingdom using a population-based incident cohort spanning the period 1940-1 991. | Variations in reported diabetes mortality could arise from true differences in case fatality rates as well as from diverse national practices in the certification of deaths. WHO MSVDD allowed for a geographic comparison of diabetes deaths, with comparable groups of patients. |  |  |
| Methods | | |  |  |  |  |  |
| Study design | 4 | Present key elements of study design early in the paper | The cohort has been constituted from various registers compiled in the UK since the early 1970s (Table 1). Overall, we were notified of over 29 000 patients with diabetes diagnosed under the age of 50 years, but entry into the present analysis was restricted to patients with insulin-treated diabetes who were diagnosed under 30 years, were under the age of 85 at the time of entry to follow-up, and had been registered before July 1993. | All children developing IDDM who were diagnosed under the age of 17 years and resident in the county of Leicestershire at the time of diagnosis between 1 January  1940 and 31 December 1989 were studied. | See 6 |  |  |
| Setting | 5 | Describe the setting, locations, and relevant dates, including periods of recruitment, exposure, follow-up, and data collection | See 6 | See 6 | See 6 |  |  |
| Participants | 6 | (*a*) Give the eligibility criteria, and the sources and methods of selection of participants. Describe methods of follow-up | 23752 patients; any age, in the UK, from 1972-1993; Diagnosed with Diabetes; <30; selected from BDA register; Leicestershie, Dorset and Oxford register; Scottish register; Northern Ireland register; Followed up Feb-97 via NHSCR; CSA files; | 845 patients, under 17 yrs; from 1940-1989; Diagnosed with diabetes type 1; selected via Central Diabetic Register; clinician-based register; hospital records; followed up 31-Dec-1991 through Clinical records; death certificates | 4713 patients; any age; UK; Switzerland; Warsaw; Berlin; Zagreb; Hong Kong; Tokyo; Havana; Oklahoma; Diagnosed with Diabetes; identified via WHO Multinational Study of Vascular Disease in Diabetes (1978-1985); followed up through Death Certificate; Clinical Information followed up on given on 1-Jan-1988 |  |  |
|  |  | (*b*) For matched studies, give matching criteria and number of exposed and unexposed | Not reported | Not reported | Not reported |  |  |
| Variables | 7 | Clearly define all outcomes, exposures, predictors, potential confounders, and effect modifiers. Give diagnostic criteria, if applicable | Age; Sex | Age; Sex | Age; Sex |  |  |
| Data sources/ measurement | 8* | For each variable of interest, give sources of data and details of methods of assessment (measurement). Describe comparability of assessment methods if there is more than one group | NHSCR; CSA files; | Clinical records; death certificates | Death Certificate; Clinical Information given |  |  |
| Bias | 9 | Describe any efforts to address potential sources of bias | Patients who died within 1 week of diagnosis were omitted because the component registers did not all include persons dying at diagnosis. Patients with diabetes secondary to other conditions were also excluded, since their mortality depends on the disease causing the diabetes as well as the diabetes itself. | Not reported | Excluded patient with no 'last known alive' date; |  |  |
| Study size | 10 | Explain how the study size was arrived at | From cohort size | From cohort size | From cohort size |  |  |
| Quantitative variables | 11 | Explain how quantitative variables were handled in the analyses. If applicable, describe which groupings were chosen and why | Not reported | Not reported | Not reported |  |  |
| Statistical methods | 12 | (*a*) Describe all statistical methods, including those used to control for confounding | SMR; Poisson distribution; | Cox-proportional hazards; Kaplan-Meier | SMR; |  |  |
|  |  | (*b*) Describe any methods used to examine subgroups and interactions | Not reported | Not reported | Not reported |  |  |
|  |  | (*c*) Explain how missing data were addressed | Not reported | Not reported | Not reported |  |  |
|  |  | (*d*) If applicable, explain how loss to follow-up was addressed | Excluded untraced patient; missing entries patient excluded; diabetes as secondary cause of death excluded | Used last available data | Excluded patient with no 'last known alive' date; |  |  |
|  |  | (*e*) Describe any sensitivity analyses | Not reported | Not reported | Not reported |  |  |
| Results | | |  |  |  |  |  |
| Participants | 13* | (a) Report numbers of individuals at each stage of study—eg numbers potentially eligible, examined for eligibility, confirmed eligible, included in the study, completing follow-up, and analysed | 23752 patients initial; 949 patient deaths | 845 patients initial; 44 patient deaths | 4713 patients initial;1099 patient deaths; |  |  |
|  |  | (b) Give reasons for non-participation at each stage | Death | Death | Death |  |  |
|  |  | (c) Consider use of a flow diagram |  |  |  |  |  |
| Descriptive data | 14* | (a) Give characteristics of study participants (eg demographic, clinical, social) and information on exposures and potential confounders | See 6 | See 6 | See 6 |  |  |
|  |  | (b) Indicate number of participants with missing data for each variable of interest | Not reported | Not reported | Not reported |  |  |
|  |  | (c) Summarise follow-up time (eg, average and total amount) | 20 years | 50 years | 10 years |  |  |
| Outcome data | 15* | Report numbers of outcome events or summary measures over time | 12 outcomes | 10 outcomes | 18 outcomes |  |  |
| Main results | 16 | (*a*) Give unadjusted estimates and, if applicable, confounder-adjusted estimates and their precision (eg, 95% confidence interval). Make clear which confounders were adjusted for and why they were included | 949 patient deaths; mortality rates for diabetic patient exceeded gen. pop.; SMR 4.0 for females cf. 2.7 males; | 44 patient deaths; 31 yr median age; SMR fell from 981 to 238 relative to gen. pop. Over life of study | 1099 patient deaths; Cardiovascular disease responsible for 44% of Type 1 deaths and 52% of Type 2 deaths; |  |  |
|  |  | (*b*) Report category boundaries when continuous variables were categorized | Not reported | Not reported | Not reported |  |  |
|  |  | (*c*) If relevant, consider translating estimates of relative risk into absolute risk for a meaningful time period | Not reported | Not reported | Not reported |  |  |
| Other analyses | 17 | Report other analyses done—eg analyses of subgroups and interactions, and sensitivity analyses | Not reported | Not reported | Not reported |  |  |
| Discussion | | |  |  |  |  |  |
| Key results | 18 | Summarise key results with reference to study objectives | The SMRs, reflecting the risk of death relative to that in the general population, were greater for females than males at all  ages. The overall SMR for females was 4.0 (95% CI 3.6±4.4) and for males was 2.7 (95% CI 2.5±2.9) reaching a peak in females of 5.7 (4.7±7.0) at ages 20±29 and in males of 4.0 (3.1±5.0) at ages 40±49. | During the follow-up period from 1 .1 .I940 to 31.12.1991, 44 (5.2 Yo) deaths were recorded. A further 4 deaths (2 male and 2 female) occurred at the onset of  diabetes (within 24 h), all associated with ketoacidosis, with death in 2 cases occurring outside hospital care. | See 16 |  |  |
| Limitations | 19 | Discuss limitations of the study, taking into account sources of potential bias or imprecision. Discuss both direction and magnitude of any potential bias | Our own study supports this, since only 67% of the death certificates mentioned diabetes, and it was an underlying cause of death in only 36% of the cases | Although the cohort we investigated is large, the death rate is relatively low, and hence the power of the analysis is somewhat restricted. We believe that we have demonstrated convincing evidence of a marked decline in the mortality of juvenile onset IDDM for patients diagnosed in the 1940s compared to those diagnosed in the 1980s. However, although we have tended to interpret this as a gradual improvement in survival over time the data are also consistent with a large fall in mortality between the 1940s and 1950s (particularly in females) followed by a longish period of stability. In effect the study has inadequate power to discriminate between the two possibilities. | Some of the WHO MSVDD centres, such as Havana, Zagreb, Hong Kong and Berlin had little previous information available on diabetes mortality. |  |  |
| Interpretation | 20 | Give a cautious overall interpretation of results considering objectives, limitations, multiplicity of analyses, results from similar studies, and other relevant evidence | This international variation in mortality rates in patients with Type 1DM, together with variation in the underlying populations and variation with time, all emphasize the need for current mortality statistics speci®c to the UK. | This analysis of trends in mortality shows that the prognosis of childhood onset IDDM has improved substantially over the past five decades. We hope this improvement in prognosis will be reflected in lower insurance premiums for juvenile-onset IDDM in the future. Since this cohort is still relatively young further follow-up is required since larger numbers will then be at risk of renovascular disease. | Cardiovascular disease was the most common underlying cause of death, accounting for 44 % of deaths in Type I (insulin-dependent) diabetes mellitus and 52 % of deaths in Type II (non-insulin-dependent) diabetes mellitus. Renal disease accounted for 21% of deaths in Type I diabetes and 11% in Type II diabetes. For Type I diabetes, all-cause mortality rates were highest in Berlin men and Warsaw women, and lowest in London men and Zagreb women. For Type II diabetes, rates were highest in Warsaw men and Oklahoma women and lowest in Tokyo men and women. Age adjusted mortality rates and SMRs were generally higher in patients with Type I diabetes compared with those with Type II diabetes. Men and women in the Tokyo cohort had a very low excess mortality when compared with the background population. |  |  |
| Generalisability | 21 | Discuss the generalisability (external validity) of the study results | Previous studies have included comparatively few young subjects and had little scope for measuring age- and sex-specific mortality at young ages with any precision [4±8].  Results from the Pittsburgh study also showed mortality rates in males with Type 1DMexceeding those in females only at older ages. | In accord with recent reports from other countries individuals with childhood-onset IDDM demonstrate an excess mortality compared to the general population. The two-fold excess mortality for the cohort  diagnosed in the 1980s is similar to recent reports from Sweden (210; 110 to 380),'l and Norway (210; 130 to 320) regarding short-term mortality from diabetes.2" | Few international comparisons of diabetes mortality exist. The DERI study looked at mortality among patients with Type 1 diabetes from 1965-1985. There was considerable international variation in all-cause mortality among subjects from Japan, Finland and the USA. |  |  |
| Other information | | |  |  |  |  |  |
| Funding | 22 | Give the source of funding and the role of the funders for the present study and, if applicable, for the original study on which the present article is based | None reported | None reported | None reported |  |  |

|  | Item No | Recommendation | Muggeo et al, 1995 [32] | Nishimura et al, 2001 [5] | Podar et al, 2000 [16] |  |  |
| --- | --- | --- | --- | --- | --- | --- | --- |
| **Title and abstract** | 1 | (*a*) Indicate the study’s design with a commonly used term in the title or the abstract | The Verona diabetes study: a population-based survey on known diabetes mellitus prevalence and 5-year all-cause mortality | Mortality trends in type 1 diabetes - The Allegheny County (Pennsylvania) Registry 1965-1999 | Mortality in patients with childhood-onset type 1 diabetes in Finland, Estonia, and Lithuania - Follow-up of nationwide cohorts |  |  |
|  |  | (*b*) Provide in the abstract an informative and balanced summary of what was done and what was found | This population-based survey aimed to determine the prevalence of known diabetes mellitus on 31 December 1986, and to assess all-cause mortality in the subsequent 5 years (1987-1991) in Verona, Italy. In the study of prevalence, 5996 patients were identified by three independent sources: family physicians, diabetes clinics, and drug prescriptions for diabetes. Mortality was assessed by matching all death certificates of Verona in 1987-1991 with the diabetic cohort. Overall diabetes prevalence was 2.61% (95% confidence interval 2.56-2.67). Prevalence of insulin-dependent and non-insulin-dependent diabetes mellitus was 0.069% (0.059-0.078) and 2.49% (2.43-2.54), respectively. Diabetes prevalence sharply increased after age 35 years up to age 75-79, and finally declined. Prevalence was higher in men up to age 69 years, in women after age 75 years. Of the diabetic cohort 1260 patients (592 men, 668 women) died by 31 December 1991, yielding an overall standardized mortality ratio of 1.46 (CI 1.38-1.54). Even though the differences narrowed with age, mortality rates in the diabetic cohort were higher than in the non-diabetic population at all ages. Women aged 65-74 years showed observed/expected ratio higher than men (2.27, CI 1.92-2.66, vs 1.50, CI 1.30-1.72), while in other age groups the sex-related differences were not significant. Pharmacological treatment of diabetes was associated with an excess mortality, while treatment with diet alone showed an apparent protective effect on mortality (observed/expected ratio 0.73, CI 0.58-0.92) | OBJECTIVES—To investigate long-term mortality and its temporal trends as of 1 January 1999 among the 1,075 patients with type 1 diabetes (onset age <18 years, diagnosed between 1965 and 1979) who comprise the Allegheny County population-based registry. RESEARCH DESIGN AND METHODS—Overall, sex- and race-specific mortality rates per person-year of follow-up were determined. Standardized mortality ratios were also calculated. Survival analyses and Cox proportional hazard model were also used. Temporal trends were examined by dividing the cohort into three groups by year of diagnosis (1965–1969, 1970–1974, and 1975–1979). RESULTS—Living status of 972 cases was ascertained as of January 1, 1999 (ascertainment rate 90.4%). The mean duration of diabetes was 25.2 ± 5.8 (SD) years. Overall, 170 deaths were observed. The crude mortality rate was 627 per 100,000 person-years (95% CI 532–728) and standardized mortality ratio was 519 (440–602). Life-analyses by the Kaplan-Meier method indicated cumulative survival rates of 98.0% at 10 years, 92.1% at 20 years, and 79.6% at 30 years duration of diabetes. There was a significant improvement in the survival rate between the cohort diagnosed during 1965–1969 and that diagnosed during 1975–1979 by the log-rank test (P = 0.03). Mortality was higher in African-Americans than in Caucasians, but there were no differences seen by sex. The improvement in recent years was seen in both ethnic groups and sexes. CONCLUSIONS—An improvement in long-term survival was observed in the more recently diagnosed cohort. This improvement is consistent with the introduction of HbA1 testing, home blood glucose monitoring, and improved blood pressure therapy in the 1980s. | Objective - To assess mortality of population-based cohorts of childhood-onset type 1diabetic patients from the Eastern European countries of Estonia and Lithuania and compare this information with recent data from Finland. RESEARCH DESIGN AND METHODS— Estonian (n = 518) and Finnish (n = 5,156) type 1 diabetic cohorts were diagnosed between 1980 and 1994, and the Lithuanian (n = 698) cohort was diagnosed between 1983 and 1994. The mortality of these cohorts was determined in 1995. Life-table analysis, Cox survival analysis with covariates, and standardized mortality ratios (SMRs) were used. Causes of death were analyzed. Results — Survival after 10 years duration of type 1 diabetes was similar in Estonia (94.3%) and Lithuania (94.0%), but much higher in Finland (99.1%). In the Cox survival analysis with covariates, the country of origin and age at diagnosis were found to be significant predictors of mortality. The SMR for the Estonian cohort was 4.35 (95% CI 2.25–7.61), the highest for the Lithuanian cohort was 7.55 (4.89–11.15), and the lowest for the Finnish cohort was 1.62 (1.10–2.28). The most common cause of death in Estonia and Lithuania was diabetic ketoacidosis (DKA), and in Finland, it was violent causes. No deaths from late complications of diabetes have been documented so far in any of the three countries.  Conclusions— Our results demonstrate a high rate of short - term deaths due to DKA and inferior survival of childhood-onset type 1 diabetic patients in Estonia and Lithuania compared with Finland. In Finland, the survival of childhood-onset type 1 diabetic patients has improved and is only slightly inferior to that of the background population. |  |  |
| Introduction | | |  |  |  |  |  |
| Background/rationale | 2 | Explain the scientific background and rationale for the investigation being reported | For the majority of southern European countries data on the epidemiology of diabetes mellitus and related mortality are not conclusive. In this area, the overall diabetes prevalence has an apparently wide variability, ranging between i to 23 %, a difference reflecting methodological rather than geographical differences [1]. In Italy studies carried out using similar methodology recently reported that known diabetes prevalence ranges from 2.2-2.5 % [2, 3]. | The Diabetes Epidemiology Research International (DERI) group conducted an international study comparing the mortality (as of 1 January 1990) of patients diagnosed between 1965 and 1979 in population-based registries from four countries: U.S. (Allegheny County, PA), Finland, Japan, and Israel. The mortality in Allegheny County was five times higher than that of the general population (3,4,5) | However, the quality of life and the life expectancy of type 1 diabetic patients still lag behind that of the background population (1). Even today, type 1diabetes leads to a two- to tenfold excess risk of mortality in developed countries, where as in developing countries, a large proportion of type 1 diabetic patients die within a few years of diagnosis (1,2). |  |  |
| Objectives | 3 | State specific objectives, including any prespecified hypotheses | The aim of the present study was to determine the prevalence of known diabetes and to assess diabetes related 5-year all-cause mortality in Verona, a midsize town of north-east Italy. | However, very limited information is available in the U.S. regarding time trends in mortality with duration of diabetes of >10 years for patients diagnosed in recent years (9). This is particularly important because medical care improved greatly with the advent of self-monitoring blood glucose (10), HbA1c (11), and angiotensin-converting enzyme inhibitor use (12) in recent years. Therefore, the current study assessed the long-term mortality and temporal trends in prognosis for the subjects diagnosed between 1965 and 1979 with follow-up of at least 19 years. Differences in ethnic group (African-American versus Caucasian) and sex were also examined. | The purpose of the present study was to estimate the mortality of population-based cohorts of childhood-onset type 1 diabetic patients from the Eastern European countries of Estonia and Lithuania and compare it with the latest data from Finland. |  |  |
| Methods | | |  |  |  |  |  |
| Study design | 4 | Present key elements of study design early in the paper | Designed to ascertain all known diabetes  cases in the area of Verona, by using three independent sources: family physicians, diabetes clinics, and a database of prescriptions for antidiabetic drugs and diagnostic and therapeutic devices.  The rationale on which the study is based is that an accurate estimate of prevalence of known diabetes can be assessed only by using simultaneously more than one source for identification of diabetic subjects [2, 3]. | Subjects were identified from the type 1 diabetes incidence registry in Allegheny County, PA, which was developed through periodic review of hospital records and validated by contact with pediatricians in the community (13). This is the same cohort as studies in earlier DERI reports (3,4,5) and has been fully described previously (3,4,5,13) with the degree of ascertainment estimated to be >95% (13). | In all three countries, the diagnostic criteria for type 1 diabetes were those defined by the World Health Organization DIAMOND project, as follows: 1) diagnosis of diabetes; 2) placed on insulin before age 15; and 3) permanent residency in the country at the time of the first insulin administration (2). |  |  |
| Setting | 5 | Describe the setting, locations, and relevant dates, including periods of recruitment, exposure, follow-up, and data collection | The area where the Verona Diabetes Study was carried out corresponds to the Social Health Unit of Verona (Verona SHU). The population of this area consisted of 301,519 inhabitants on 31 December 1986. | See 6 | See 6 |  |  |
| Participants | 6 | (*a*) Give the eligibility criteria, and the sources and methods of selection of participants. Describe methods of follow-up | 5996 patients; Diagnosis of Diabetes <35 yrs; started insulin treatment <2 yrs; Verona, Italy; from 1987-1991; identified via Social Health Unit of Verona ; Juvenile Diabetes Centre; followed up via Mortality records Verona SHU on 31-Dec-1991 | 1075 patients; any age; Inclusion criteria were a diagnosis of diabetes before 18 years of age between 1 January 1965 and 31 December 1979, residence in Allegheny County, PA, at onset, and administration of insulin beginning at the time of diagnosis; sourced through Allegheny Country registry; followed up 1-jan-1999 through National Death Registry | 518 patients; Estonia; 5156 patients; Finland; 698 patients; Lithuanian; 1980-1994; Diagnosis of diabetes; placed on insulin <15 yr; permanent resident of country; followed up via National Death Registry; Currency Exchange Lists on 1-Jul-1995 |  |  |
|  |  | (*b*) For matched studies, give matching criteria and number of exposed and unexposed | Not reported | Not reported | Not reported |  |  |
| Variables | 7 | Clearly define all outcomes, exposures, predictors, potential confounders, and effect modifiers. Give diagnostic criteria, if applicable | Age; Sex | Age; Sex | Age; Sex |  |  |
| Data sources/ measurement | 8* | For each variable of interest, give sources of data and details of methods of assessment (measurement). Describe comparability of assessment methods if there is more than one group | Mortality records Verona SHU | National Death Registry | National Death Registry |  |  |
| Bias | 9 | Describe any efforts to address potential sources of bias | Not reported | Subjects excluded if diabetes was developed from a secondary cause. | Not reported |  |  |
| Study size | 10 | Explain how the study size was arrived at | From cohort size | From cohort size | From cohort size |  |  |
| Quantitative variables | 11 | Explain how quantitative variables were handled in the analyses. If applicable, describe which groupings were chosen and why | Not reported | Not reported | Not reported |  |  |
| Statistical methods | 12 | (*a*) Describe all statistical methods, including those used to control for confounding | t-test; SMR; Byar's Approximation; Possion distribution | chi squared test; Kaplan-Meier; Log-rank; SMR; Cox proportional hazard; Poisson distribution | Life-table analysis; Cox survival analysis; SMR; Possion distribution |  |  |
|  |  | (*b*) Describe any methods used to examine subgroups and interactions | Not reported | Not reported | Not reported |  |  |
|  |  | (*c*) Explain how missing data were addressed | Not reported | Not reported | Not reported |  |  |
|  |  | (*d*) If applicable, explain how loss to follow-up was addressed | Not reported | Not reported | Not reported |  |  |
|  |  | (*e*) Describe any sensitivity analyses | Not reported | Not reported | Not reported |  |  |
| Results | | |  |  |  |  |  |
| Participants | 13* | (a) Report numbers of individuals at each stage of study—eg numbers potentially eligible, examined for eligibility, confirmed eligible, included in the study, completing follow-up, and analysed | 5996 patients initial; | 1075 patients initial; 170 patients deceased | 518 estonian patients; 5156 finnish patients; 698 lithuanian patients |  |  |
|  |  | (b) Give reasons for non-participation at each stage | Death | Death | Death |  |  |
|  |  | (c) Consider use of a flow diagram |  |  |  |  |  |
| Descriptive data | 14* | (a) Give characteristics of study participants (eg demographic, clinical, social) and information on exposures and potential confounders | See 6 | See 6 | See 6 |  |  |
|  |  | (b) Indicate number of participants with missing data for each variable of interest | Not reported | Not reported | Not reported |  |  |
|  |  | (c) Summarise follow-up time (eg, average and total amount) | 5 years | 25 years | 15 years |  |  |
| Outcome data | 15* | Report numbers of outcome events or summary measures over time | 11 outcomes | 13 outcomes | 10 outcomes |  |  |
| Main results | 16 | (*a*) Give unadjusted estimates and, if applicable, confounder-adjusted estimates and their precision (eg, 95% confidence interval). Make clear which confounders were adjusted for and why they were included | SMR 1.46 cf. gen pop; pharmacological treatment associated with excess mortality; diet-alone showed protective effect on mortality | 170 patient deaths; SMR 519; African-American mortality>Caucasians; no gender difference; improvement in group diagnosed '75-'79 cf. '65-'69 | SMR 4.35 cf gen. pop; mean death age 16.4; SMR 1.62 cf gen. pop; mean death age 18.7; SMR 7.55 cf gen. pop; mean death age 14.7 |  |  |
|  |  | (*b*) Report category boundaries when continuous variables were categorized | Not reported | Not reported | Not reported |  |  |
|  |  | (*c*) If relevant, consider translating estimates of relative risk into absolute risk for a meaningful time period | Not reported | Not reported | Not reported |  |  |
| Other analyses | 17 | Report other analyses done—eg analyses of subgroups and interactions, and sensitivity analyses | Not reported | Not reported | Not reported |  |  |
| Discussion | | |  |  |  |  |  |
| Key results | 18 | Summarise key results with reference to study objectives | The main findings of the Verona Diabetes Study are: 1) an overall prevalence of known diabetes of 2.61% (CI 2.56-2.67) on 31 December 1986; 2) an overall all-cause SMR of 1.46 (CI 1.38-1.54) in the 5 years of follow-up. This information was obtained from a well-characterized cohort of diabetic subjects living in a well-defined urban area of north-east Italy. | In accordance with recent reports from developed countries, the mortality in Allegheny Country, PA, was higher than that of the general population with an overall SMR of 519 (95% CI 440–672). | Survival of the patients in the Estonian and Lithuanian cohorts was quite similar, as expected. Both countries were part of the former Soviet Union and subject to similar health care practices. The Finnish cohort had better survival compared with that in the two Baltic countries for several reasons. Type 1 diabetes is much more common in Finland than in the Baltic states. Thus, the society, health care system, and physicians in Finland are much more experienced in taking care of diabetic patients. Second, a higher living standard in Finland has enabled enough resources for self-monitoring and education of patients. |  |  |
| Limitations | 19 | Discuss limitations of the study, taking into account sources of potential bias or imprecision. Discuss both direction and magnitude of any potential bias | However, it should be mentioned that in young age groups the evaluation of mortality risk is hampered by the generally low number of observed and expected deaths. Moreover, the life expectancy in young diabetic patients has dramatically increased over the last 20 years, and this might strongly contribute to differences between recent as compared to earlier studies. | 10% of cases living status not determined; | Not reported |  |  |
| Interpretation | 20 | Give a cautious overall interpretation of results considering objectives, limitations, multiplicity of analyses, results from similar studies, and other relevant evidence | Two studies with similar characteristics have been recently carried out in Scotland [24, 25] and agreed with our finding that diabetic patients have a mortality risk approximately 50 % higher than non-diabetic subjects. Earlier investigations reported a two [26] or threefold [27] increase in mortality risk in diabetes. This discrepancy could reflect an improvement in diabetes prognosis from the 1960s, to the 1970s and into the 1980s, when the two Scottish studies [24, 25] and the Verona Diabetes Study were carried out. | The reasons for a higher mortality in the U.S. may relate to the costly health care system for diabetes (5); the high financial burden may keep patients from frequent contact with physicians. In Finland and Norway, where mortality was reported to be lower, free medical care is provided for type 1 diabetes. Greater contact with a diabetes specialist care team has also been suggested to be related to a reduced risk of death in Pittsburgh (18). Another component of the higher mortality in the U.S. is the higher mortality of African-Americans, which has previously been reported as a twofold excess mortality in the same cohort (19). | The short-term survival of childhood-onset type 1 diabetic patients in Estonia and Lithuania is inferior compared with that in Finland. We can only speculate on the cause of our observations, but data suggest the importance of patient education and self-monitoring as possible means to reducing the excess mortality of type 1 diabetic patients in the Baltic states and perhaps in other Eastern European countries. In Finland, the survival of childhood-onset type 1 diabetic patients has improved and is only slightly inferior to that of the background population. |  |  |
| Generalisability | 21 | Discuss the generalisability (external validity) of the study results | When extending the comparison to results obtained in other southern European countries, the great methodological differences among the various surveys must be taken into account. They make comparison difficult and lead to a diabetes prevalence ranging from 1% to 23 % [1]. In addition to methodology, also demographic and geographic differences could account for the different prevalence rates of known diabetes found in northern Europe [11-14].  However, it is noteworthy that recent data from Finland [15, 16] are similar to those in our study, despite these studies being based on diabetes registers. | Likewise, a study from Leicestershire, U.K., followed 845 type 1 diabetes patients diagnosed between 1940 and 1989 and reported a decrease in the SMR from 938 in the 1940s to 238 in the 1980s (8), although clearly this decrease was dated from an earlier time point. However, in a more recent study (cases diagnosed between 1973 and 1982 compared with 1988), the SMR in Norway has also been reported to have decreased from 1,908 to 207 (6). Although our results are somewhat worse than these European data, differences in methodology (including our longer follow-up) and populations studied make comparisons difficult. | The SMRs were 1.93 and 1.62 in the DERI Study (11) and in our study, respectively; the SMR is now approaching that of the background population (12). |  |  |
| Other information | | |  |  |  |  |  |
| Funding | 22 | Give the source of funding and the role of the funders for the present study and, if applicable, for the original study on which the present article is based | grants No. 86.01873.56, 87.00374.56, 91.00400.PF40, 92.00337.PF40 and  93.00421.PF40 from the Italian Consiglio Nazionale delle Ricerche (CNR) (INV 953541) and by 1986 to 1994 contributions  from the Ministero dell' Universit?a e della Ricerca Scientificae Tecnologica (MURST). | Imperial Boshi Aiikkukai Foundation; National Institutes of Health Grant DK-34818 | European Community Contract BMH1 CT92 0043; Estonian Science Foundation Grant 4325 |  |  |

|  | Item No | Recommendation | Raymond et al, 1995 [33] | Riley et al, 1995 [34] | Roberts et al, 2004 [35] |  |  |
| --- | --- | --- | --- | --- | --- | --- | --- |
| **Title and abstract** | 1 | (*a*) Indicate the study’s design with a commonly used term in the title or the abstract | Insulin treated diabetes mellitus: causes of death determined from record linkage of population based registers in Leicestershire, UK | The 1984 Tasmanian insulin treated diabetes mellitus prevalence cohort: an eight and a half year mortality follow-up investigation | Mortality in young people admitted to hospital for diabetes: database study |  |  |
|  |  | (*b*) Provide in the abstract an informative and balanced summary of what was done and what was found | STUDY OBJECTIVE: Analyses of causes of mortality in people with diabetes using data form death certificates mentioning diabetes provide unreliable estimates of mortality. Under-recording of diabetes as a cause on death certificates has been widely reported, ranging from 15-60%. Using a population based register on people with diabetes and linking data from another source is a viable alternative. Data from the Office of Population Censuses and Surveys (OPCS) are the most acceptable mortality data available for such an exercise, as direct comparison with other published mortality rates is then possible. DESIGN: A locally maintained population-based mortality register and all insulin-treated diabetes mellitus cases notified to the Leicestershire diabetes register (n = 4680) were linked using record linkage software developed in-house (Lynx). This software has been extensively used in a maintenance and update cycle designed to maximise accuracy and minimise duplication and false registration on the diabetes register. Deaths identified were initially coded locally to the International Classification of Diseases, 9th revision (ICD9), and later a linkage was performed to use official OPCS coding. Mortality data identified by the linkage was indirectly standardised using population data for Leicestershire for 1991. Standardised mortality ratios (SMR) were estimated, with 95% confidence intervals. Insulin dependent diabetes (IDDM) was defined as diabetes diagnosed before age 30 years with insulin therapy begun within one year of diagnosis. All other types were considered non-insulin dependent diabetes (NIDDM). Analyses were performed for the whole sample and then for the NIDDM subgroup. Results from these analyses were similar and therefore only whole group analyses are presented. MAIN RESULTS: A total of 370 deaths were identified for the period of 1990-92 inclusive - 56% were in men and 44% in women, median age (range) 71 years (12-94). Approximately 90% of deaths were subjects with NIDDM. Diabetes was mentioned on 215 (58%) death certificates. The all causes SMRs were significantly raised for men and women for all ages less than 75 years. Ischaemic heart disease (ICD9) rubrics 410-414) accounted for 146 (40%) deaths - 41% of male and 38% of female deaths. Male and female SMRs were significantly raised for the age groups 45-64, 65-74, and 75-84 years. Cerebrovascular disease (ICD9 rubrics 430-438) accounted for 39 (10%) deaths and the SMR for women the external causes of death (ICD9 rubrics E800-E999) were also significantly raised overall and in age groups 15-44 and 45-64 years. This was not true for men, although numbers of deaths in this category were small for both men (4) and women (9). CONCLUSION: Record linkage has been used successfully to link two local, population based registers. This has enabled an analysis of mortality in people with diabetes to be performed which overcomes the problems associated with using as a sample, death certificates where diabetes is mentioned. The mortality rates and SMRs estimated should more accurately reflect the true rates than would be possible using other methods. The persisting excess mortality identified for people with diabetes is of a similar magnitude and attributable to similar causes as has been reported elsewhere in population based studies. | Total mortality and underlying cause of death were examined in a population-based prevalence cohort (n = 1232) of Tasmanians with insulin-treated diabetes mellitus. Eight and a half years after the establishment of the registry, the cause of death based on death certificate information was determined for the overall cohort and for three classification groups of insulin-treated diabetes: Group A--childhood-onset IDDM cases; Group B--adult-onset IDDM cases; and Group C--adult-onset insulin-treated NIDDM cases. A total of 378 deaths occurred, providing an overall SMR of 2.2 (95% CI 2.0-2.4) compared to the Tasmanian population. Diabetic females experienced a higher SMR (2.6, 95% CI 2.3-3.0) than diabetic males (1.9, 95% CI 1.6-2.2). The all-cause SMRs for the diabetic classification groups were 4.6 (95% CI 3.4-6.1) in Group A, 1.8 (95% CI 1.5-2.1) in Group B, and 2.2 (95% CI 1.9-2.6) in Group C. After adjusting for age, gender and duration of diabetes, the mortality in Group C was significantly higher compared to Group B (odds ratio 1.6, 95% CI 1.2-2.3). This study indicates that people with childhood-onset IDDM experience 4.6 times the death rate compared to the Tasmanian population and that the excess mortality is most pronounced in females. | None supplied |  |  |
| Introduction | | |  |  |  |  |  |
| Background/rationale | 2 | Explain the scientific background and rationale for the investigation being reported | Previous studies have reported an overall excess mortality for people with diabetes.  Ischaemic heart disease has been implicated as a particular contributor to excess mortality as has cerebrovascular disease. There have been conflicting reports of cancer mortality. Different study methods and different samples make comparison of results between studies difficult. | It is well known that population estimates of diabetes mortality based on death certificates alone will greatly underestimate the true number of deaths [1,2] because the presence of diabetes is not always noted on the death certificate. For this reason, the most reliable estimates of mortality due to diabetes come from well defined cohorts which have been followed prospectively. | People with type 1 diabetes mellitus have an increased risk of death at a young age. |  |  |
| Objectives | 3 | State specific objectives, including any prespecified hypotheses | This study aimed to investigate the possibility of using record linkage and a population based register of people with diabetes to detect deaths, and to examine  causes of deaths and patterns | This investigation examines the mortality patterns in a population-based cohort of insulin-treated people in Tasmania who were identified as using insulin on May 1, 1984 and then followed prospectively for eight and a half years. The study of a cohort of insulin-treated diabetics has the advantage of being subject to little misclassification bias and insulin prescription data can be used to measure cohort completeness. Mortality rates were assessed within defined categories of insulin-treated diabetes, based on age at onset and time between diabetes diagnosis and commencement of insulin therapy. | We aimed to quantify this risk in a population of people admitted to hospital for type 1 diabetes and to determine whether mortality in  the three year period after admission has fallen in recent years. |  |  |
| Methods | | |  |  |  |  |  |
| Study design | 4 | Present key elements of study design early in the paper | A population based register of people with  insulin treated diabetes was established in  Leicestershire during 1983-84, when the conversion to U100 insulin was implemented. | The study cohort consisted of  1232 subjects listed on the Tasmanian Insulin-Treated Diabetes Register who were resident in Tasmania and using insulin on May 1, 1984 | From 1968 to 1996, for people aged under 30 years in the area covered by the Oxford record linkage study (population of 0.9 million in 1968, 1.9 million in 1974, and 2.5 million in 1987) we analysed all NHS hospital admissions for diabetes mellitus (ICD-9, code 250) in which that condition was coded as the principal diagnosis. The database included linkage to death certificate data to 1999. |  |  |
| Setting | 5 | Describe the setting, locations, and relevant dates, including periods of recruitment, exposure, follow-up, and data collection | See 6 | See 6 | See 6 |  |  |
| Participants | 6 | (*a*) Give the eligibility criteria, and the sources and methods of selection of participants. Describe methods of follow-up | 4680 patients; over 15 yrs; from the UK; from 1990-1992; Diabetes diagnosed <30yr; insulin treatment started within 12 mths; identified through Leicestershire mortality list; population registries; followed up as of 30-dec-1992 through Mortality Register | 1232 patients; over 15 yrs old; Diagnosis of Diabetes; resident of Tasmina; on insulin as of 1-May-1984; selected via Tasmanian Insulin-Treated Diabetes Register from 1984-1992; followed up in 1992 through Electoral roll; personal contact; telephone contact | 4992 patients; UK; Diagnosis of Diabetes; <30 yr; from 1968-1996; identified through Oxford Record Linkage Study; followed up 1999 through Death Certificates |  |  |
|  |  | (*b*) For matched studies, give matching criteria and number of exposed and unexposed | Not reported | Not reported | Not reported |  |  |
| Variables | 7 | Clearly define all outcomes, exposures, predictors, potential confounders, and effect modifiers. Give diagnostic criteria, if applicable | Age; Sex | Age; Sex | Age; Sex |  |  |
| Data sources/ measurement | 8* | For each variable of interest, give sources of data and details of methods of assessment (measurement). Describe comparability of assessment methods if there is more than one group | Mortality Register | Electoral roll; personal contact; telephone contact | Death Certificates; |  |  |
| Bias | 9 | Describe any efforts to address potential sources of bias | One patient death <15 excluded due to CF | Unknown datapoints resulted in patient removed from cohort | Not reported |  |  |
| Study size | 10 | Explain how the study size was arrived at | Cohort size | Cohort size | Cohort size |  |  |
| Quantitative variables | 11 | Explain how quantitative variables were handled in the analyses. If applicable, describe which groupings were chosen and why | Not reported | Not reported | Not reported |  |  |
| Statistical methods | 12 | (*a*) Describe all statistical methods, including those used to control for confounding | Fisher's exact tests; chi squared test | SMR; multivariate logistic regression analysis | SMR; |  |  |
|  |  | (*b*) Describe any methods used to examine subgroups and interactions | Not reported | Not reported | Not reported |  |  |
|  |  | (*c*) Explain how missing data were addressed | Not reported | Not reported | Not reported |  |  |
|  |  | (*d*) If applicable, explain how loss to follow-up was addressed | Not reported | Not reported | Not reported |  |  |
|  |  | (*e*) Describe any sensitivity analyses | Not reported | Not reported | Not reported |  |  |
| Results | | |  |  |  |  |  |
| Participants | 13* | (a) Report numbers of individuals at each stage of study—eg numbers potentially eligible, examined for eligibility, confirmed eligible, included in the study, completing follow-up, and analysed | 4680 patients initial; 370 patient deaths | 1232 patients initially; 378 patient deaths; | 4992 patients initially; 58 patient deaths; |  |  |
|  |  | (b) Give reasons for non-participation at each stage | Not reported | Not reported | Not reported |  |  |
|  |  | (c) Consider use of a flow diagram |  |  |  |  |  |
| Descriptive data | 14* | (a) Give characteristics of study participants (eg demographic, clinical, social) and information on exposures and potential confounders | See 6 | See 6 | See 6 |  |  |
|  |  | (b) Indicate number of participants with missing data for each variable of interest | Not reported | Not reported | Not reported |  |  |
|  |  | (c) Summarise follow-up time (eg, average and total amount) | 2 years | 8 years | 28 years |  |  |
| Outcome data | 15* | Report numbers of outcome events or summary measures over time | 6 outcomes | 10 outcomes | 12 outcomes |  |  |
| Main results | 16 | (*a*) Give unadjusted estimates and, if applicable, confounder-adjusted estimates and their precision (eg, 95% confidence interval). Make clear which confounders were adjusted for and why they were included | 370 patient deaths; SMR sig. increased for patient <75 yr; 40% of deaths due to IHR | 378 patient deaths; SMR 2.2 cf gen. pop; childhood-onset IDDM have 4.6x death rate cf. gen. pop | 58 patient deaths; SMR 8.5 cf gen pop; 11.7% deaths due to suicide |  |  |
|  |  | (*b*) Report category boundaries when continuous variables were categorized | Not reported | Not reported | Not reported |  |  |
|  |  | (*c*) If relevant, consider translating estimates of relative risk into absolute risk for a meaningful time period | Not reported | Not reported | Not reported |  |  |
| Other analyses | 17 | Report other analyses done—eg analyses of subgroups and interactions, and sensitivity analyses | Not reported | Not reported | Not reported |  |  |
| Discussion | | |  |  |  |  |  |
| Key results | 18 | Summarise key results with reference to study objectives | Total of 370 deaths during the years 1990-92 were identified by linking the diabetes register and the local mortality list - 163 (44%) were in females and 207 (56%) in males. One female death at age 12 years from cystic fibrosis, was excluded from the main analyses as this was the only recorded death at age less than 15 years. The median age at death was 71 years (range 12-94). | We have observed a two-fold excess of mortality in a population-based prevalence cohort of Tasmanians with insulin-treated diabetes mellitus compared to the Tasmanian population. Our study cohort is diverse and comprises individuals with various clinical types of diabetes over a wide range of ages. | There were 4992 admissions (2603 (52.1%) male) for diabetes among people aged under 30 years. There were 58 deaths during the three year follow up period (SMR 8.5; 95% CI 6.5 to 10.8; table), including 32 in the first year (14.1; 9.6 to 19.4) and 15 during the first hospital admission. Standardised mortality ratios were 27.9 (14.8 to 45.2) at one year and 12.9 (7.6 to 19.5) at three years for the 1010 patients who had been recorded at admission as having diabetic ketoacidosis or coma. |  |  |
| Limitations | 19 | Discuss limitations of the study, taking into account sources of potential bias or imprecision. Discuss both direction and magnitude of any potential bias | The under-reporting of diabetes on death certificates observed in this study confirms the problems of using death certificates that record diabetes as the underlying cause of death to investigate diabetes related mortality. In this study, diabetes was significantly more likely to be entered on the death certificates of women than men. While this finding has been reported previously, the difference (48% v 24% respectively, p=0-08) was not significant. An explanation for the discrepancy has been suggested - men are more likely to have their deaths attributed to heart disease because of the known risk for men, while other reasons may be sought for women. | Due to data collection methods, no BMI; anti-GAD results were recorded. | None reported |  |  |
| Interpretation | 20 | Give a cautious overall interpretation of results considering objectives, limitations, multiplicity of analyses, results from similar studies, and other relevant evidence | Record linkage has been used successfully to link two local, population based registers. This has enabled an analysis of mortality in people with diabetes to be performed which overcomes the problems associated with using as a sample, death certificates where diabetes is mentioned. The mortality rates and SMRs estimated should more accurately reflect the true rates than would be possible using other methods. The persisting excess mortality identified for people with diabetes is of a similar magnitude and attributable to similar causes as has been reported elsewhere in population based studies. | Diabetes was not mentioned on the death certificates of 27% of the deceased subjects, a similar proportion as noted in another cohort of diabetic Australians [2] and in Pittsburgh [9]. The proportion of under-reporting varied from 16%.in Group A (childhood-onset IDDM) to 30% in Group B (adult-onset IDDM). This underscores the fact that death certificates alone are unsuitable for monitoring diabetes mortality, even for the most easily recognised form of this disease: childhood-onset IDDM [27]. | Young people admitted to hospital for diabetes have an increased risk of death in the following three years, not only from natural causes but also from suicide. Although, in absolute terms, death in young people with diabetes is uncommon, standardised mortality  ratios showed that death within three years of hospital admission was nine times more common than in the general population. |  |  |
| Generalisability | 21 | Discuss the generalisability (external validity) of the study results | In this study, an estimated excess mortality  of 50% was found for men and one of 80% for women. Differing methods and sample compositions necessitate caution in interpretation of comparisons with other studies reporting higher or lower excesses. | Compared to similar diabetic cohorts, the overall excess mortality in Tasmanians with insulin-treated diabetes (SMR 2.2) was less than the excess reported in Wisconsin (7.5) [6], but reasonably close to observations in Southhampton, UK (1.75)  [7] and Copenhagen, Denmark (3.6) [8]. Among the diabetes classification groups, excess mortality as measured by SMR was highest in Group A (4.6), followed by Group C (2.2), and Group B (1.8). The SMRs in our older onset groups (Groups B and C) are very similar to those found in the ten studies reviewed by Panzram [8]. However, the excess mortality in cases classified as young-onset IDDM (Group A) was higher than reported in Sweden (2.1) [13] or Israel (3.2) | Other studies have reported improvements in  prognosis in recent decades for people with type 1 diabetes.1 5 We found no appreciable improvement, however, in young people admitted to hospital for diabetes in the past 30 years. Because methods for glycaemic control and the delivery of insulin therapy have improved over time, the proportion of people  admitted with diabetes whose condition is difficult to control is unlikely to have increased. Survival of young people with type 1 diabetes whose disease was serious enough to warrant admission is therefore not likely to have improved much. |  |  |
| Other information | | |  |  |  |  |  |
| Funding | 22 | Give the source of funding and the role of the funders for the present study and, if applicable, for the original study on which the present article is based | Not reported | Leon Mow Trust; RHH Research Trust; Novo Nordisk Australia; Diabetes Australia Research Trust; Juvenile Diabates Foundational International | The Oxford record linkage study was a project of the former Oxford Regional Health Authority. The Unit of Health-Care Epidemiology is funded by the Department of Health to analyse the linked data. |  |  |

|  | Item No | Recommendation | Sator & Dahlquist, 1995 [36] | Skirvarhaug et al, 2006 [37] | Soedamah-Muthu et al, 2006 [38] |  |  |
| --- | --- | --- | --- | --- | --- | --- | --- |
| **Title and abstract** | 1 | (*a*) Indicate the study’s design with a commonly used term in the title or the abstract | Short-term mortality in childhood onset insulin-dependent diabetes mellitus: a high frequency of unexpected deaths in bed | Long-term mortality in a nationwide cohort of childhood-onset type 1 diabetic patients in Norway | All-cause mortality rates in patients with type 1 diabetes mellitus compared with a non-diabetic population from the UK general practice research database, 1992-1999 |  |  |
|  |  | (*b*) Provide in the abstract an informative and balanced summary of what was done and what was found | Mortality and the causes of death have been studied in a population-based cohort of 4919 childhood onset IDDM cases. Enrolment began in 1977 and at the time of study there had been a maximum duration of disease of 13.5 years, with a total of 33,721 person years at risk. Survival status was ascertained by linkage to the Swedish Cause-of-Death register. Death certificates, autopsy protocols, and hospital records were scrutinized for classification of causes of deaths. Twenty males and 13 females with IDDM died before the age of 28.5 years. This corresponds to a Standardized Mortality Rate for age of 262% (95% confidence limits, 172-400) for the boys and 384% (95% confidence limits, 232-635) for girls. Seven patients died of ketoacidosis, four at onset of diabetes. Nine cases were found 'dead in bed', having been seen apparently healthy 1-2 days before death. One of these cases had signs of cerebral haemorrhages at autopsy and another one had signs of bite marks in the mouth, but otherwise all autopsies were normal and no evidence of alcohol or other intoxication was found. In a well-educated population with good access to inexpensive diabetes care, there is still a two- to threefold excess mortality among young onset insulin-dependent diabetic individuals including a high frequency of unexplained deaths in bed. | AIMS/HYPOTHESIS: We examined long-term total and cause-specific mortality in a nationwide, population-based Norwegian cohort of patients with childhood-onset type 1 diabetes. MATERIALS AND METHODS: All Norwegian type 1 diabetic patients who were diagnosed between 1973 and 1982 and were under 15 years of age at diagnosis were included (n=1,906). Mortality was recorded from diabetes onset until 31 December 2002 and represented 46,147 person-years. The greatest age attained among deceased subjects was 40 years and the maximum diabetes duration was 30 years. Cause of death was ascertained by reviews of death certificates, autopsy protocols and medical records. The standardised mortality ratio (SMR) was based on national background statistics. RESULTS: During follow-up 103 individuals died. The mortality rate was 2.2/1000 person-years. The overall SMR was 4.0 (95% CI 3.2-4.8) and was similar for males and females. For ischaemic heart disease the SMR was 20.2 (7.3-39.8) for men and 20.6 (1.8-54.1) for women. Acute metabolic complications of diabetes were the most common cause of death under 30 years of age (32%). Cardiovascular disease was responsible for the largest proportion of deaths from the age of 30 years onwards (30%). Violent death accounted for 28% of the deaths in the total cohort (35% among men and 11% among women). CONCLUSIONS/INTERPRETATION: Childhood-onset type 1 diabetes still carries an increased mortality risk when compared with the general population, particularly for cardiovascular disease. To reduce these deaths, attention should be directed to the prevention of acute metabolic complications, the identification of psychiatric vulnerability and the early detection and treatment of cardiovascular disease and associated risk factors. | AIMS/HYPOTHESIS: We compiled up to date estimates of the absolute and relative risk of all-cause mortality in patients with type 1 diabetes in the UK.  MATERIALS AND METHODS:  We selected patients with type 1 diabetes (n=7,713), and for each of these diabetic subjects five age- and sex-matched control subjects without diabetes (n=38,518) from the General Practice Research Database (GPRD). Baseline was 1 January 1992; subjects were followed until 1999. The GPRD is a large primary-care database containing morbidity and mortality data of a large sample representative of the UK population. Deaths occurring in the follow-up period were identified.  RESULTS:  The study comprised 208,178 person-years of follow-up. The prevalence of type 1 diabetes was 2.15/1,000 subjects in 1992 (mean age 33 years, SD 15). Annual mortality rates were 8.0 per 1,000 person-years (95% CI 7.2-8.9) in type 1 diabetic subjects compared with 2.4 per 1,000 person-years (95% CI 2.2-2.6) in those without diabetes (hazard ratio [HR]=3.7, 95% CI 3.2-4.3). The increased mortality rates in patients with type 1 diabetes were apparent across all age-bands. The HR was higher in women (HR=4.5, 95% CI 3.5-5.6 compared with non-diabetic women) than men (HR=3.3, 95% CI 2.7-4.0), such that the sex difference (p<0.0001) in mortality in the non-diabetic population was abolished (p=0.3) in the type 1 diabetic patients. The predominant cause of death in patients with type 1 diabetes was cardiovascular disease.  CONCLUSIONS/INTERPRETATION:  Despite advances in care, UK mortality rates in the past decade continue to be much greater in patients with type 1 diabetes than in those without diabetes. |  |  |
| Introduction | | |  |  |  |  |  |
| Background/rationale | 2 | Explain the scientific background and rationale for the investigation being reported | Although the introduction of insulin allowed life expectancy to increase tenfold from a median survival time of 2 years after onset of the disease,’ death rates for diabetic patients under 45 years of age are still nearly eight times of that for the general population. The main part of this excess mortality is due to long-term complications but clearly some of the excess short-term mortality is due to acute complications that may be preventable. | After insulin became available for clinical use, a marked decrease in mortality was observed. In spite of this reduction in mortality, type 1 diabetes still has a two- to ten-fold excess risk of death in developed countries [2, 3]. Diabetes mellitus is known to be under-reported on death certificates as an underlying or contributing cause of death [4–6]. Cohort studies are the best way to assess the mortality risk among diabetic patients. However, long-term follow-up studies of mortality in subjects with childhood-onset type 1 diabetes are relatively rare [2, 5, 7–11]. | Type 1 diabetes is associated with an increased risk of all-cause mortality compared with the general population. The magnitude of this risk has not been estimated very precisely. Many previous studies estimating relative risks for mortality in individuals with type 1 diabetes were small (number of patients with type 1 diabetes varied between 241 and 3,228) resulting in a wide range of estimates of relative risk for mortality (2- to 15-fold). General population rates were used for comparison, which contain those with diabetes, causing underestimation of the true relative risk |  |  |
| Objectives | 3 | State specific objectives, including any prespecified hypotheses | Since 1 July 1977 all incident cases of IDDM occurring before the age of 15 have been prospectively recorded in the Swedish Childhood Diabetes Register which covers 96-99 of all cases. To analyse in detail the early mortality of this cohort we have now followed up the survival status to the end of December 1991 of almost 5000 young people with a maximum duration of diabetes of 13.5 years corresponding to 33 721 person years at risk. | In the present study, we report total and cause-specific mortality rates during the long-term follow-up of a nationwide cohort of patients with childhood-onset type 1 diabetes in Norway. | The aim of this study is, therefore, to estimate absolute and relative all-cause mortality rates in patients with type 1diabetes compared with an age and sex matched non diabetic comparison group from the GPRD over a study period from 1992 to 1999 |  |  |
| Methods | | |  |  |  |  |  |
| Study design | 4 | Present key elements of study design early in the paper | Up to the end of December 1990, 553 boys and 455 girls in the age group 0 4 years, 899 boys and 850 girls in the age group 5-9 years, and 1201 boys and 1036 girls in the age group 10-1 4 years were reported to the register. Out of the 4994 children, parental consent to registration of the personal identification number was given in 4919 (98.5 %). A record linkage was established  for these 491 9 children to the Swedish Cause-of-Death Register and checked for mortality from 1 July 1977 up to 31 December 1990 | All new-onset cases of type 1 diabetes occurring in Norwegian children below 15 years of age during the period from 1 January 1973 to 31 December 1982 were registered retrospectively between 1985 and 1986 and included in the Norwegian Childhood Diabetes Registry [16]. The data were collected by contacting all the paediatric and medical hospital departments in Norway for information on all new patients aged 0–14 years with type 1 diabetes during the study period | This was a cohort study of type 1 diabetes and a comparison group without diabetes selected from the GPRD. The GPRD was set up in 1987 and comprises data from a large network of participating general practices that undertake to enter a minimum set of information in a standardised way onto a pre-specified database for all their patients. |  |  |
| Setting | 5 | Describe the setting, locations, and relevant dates, including periods of recruitment, exposure, follow-up, and data collection | See 6 | See 6 | See 6 |  |  |
| Participants | 6 | (*a*) Give the eligibility criteria, and the sources and methods of selection of participants. Describe methods of follow-up | 4919 patients <14 yrs; Sweden, from 1977-1990; Diagnosis of diabetes; >1 mth old; identified via Swedish Childhood Diabetes Register; followed up 31-Dec-1990 via Swedish Cause-of-Death Register | 1912 patients; <15 yrs; Norway; 1973-1982; Diagnosis of Diabetes; <15 yr; selected via Norwegian Childhood Diabetes Registry; followed up 31-Dec-2002 via Cause of Death Registry; | 7713 patients; <35: UK; 1992-1999; patient w/6 mths prior data to 1-jan-1992; <35 yrs w/diabates; patient on insulin aged <35 yrs at treatment; selected via General Practical Research Database; followed up through Death registry via GP on 1-Oct-99 |  |  |
|  |  | (*b*) For matched studies, give matching criteria and number of exposed and unexposed | Not reported | Not reported | Not reported |  |  |
| Variables | 7 | Clearly define all outcomes, exposures, predictors, potential confounders, and effect modifiers. Give diagnostic criteria, if applicable | Age; Sex | Age; Sex | Age; Sex |  |  |
| Data sources/ measurement | 8* | For each variable of interest, give sources of data and details of methods of assessment (measurement). Describe comparability of assessment methods if there is more than one group | Swedish Cause of Death Register | Cause of Death Registry | Cause of Death Registry |  |  |
| Bias | 9 | Describe any efforts to address potential sources of bias | Not reported | Exclusion if diabetes was developed from a secondary cause | Not reported |  |  |
| Study size | 10 | Explain how the study size was arrived at | Cohort Size | Cohort Size | Cohort Size |  |  |
| Quantitative variables | 11 | Explain how quantitative variables were handled in the analyses. If applicable, describe which groupings were chosen and why | Not reported | Not reported | Not reported |  |  |
| Statistical methods | 12 | (*a*) Describe all statistical methods, including those used to control for confounding | SMR | SMR; Cox regression analysis; Kaplan-Meier | Cox-proportional hazards; HR; Nelson-Alen cumilative hazards estimates; Schoenfeld method; |  |  |
|  |  | (*b*) Describe any methods used to examine subgroups and interactions | Not reported | Not reported | Not reported |  |  |
|  |  | (*c*) Explain how missing data were addressed | Not reported | Not reported | Not reported |  |  |
|  |  | (*d*) If applicable, explain how loss to follow-up was addressed | Not reported | Not reported | Not reported |  |  |
|  |  | (*e*) Describe any sensitivity analyses | Not reported | Not reported | Not reported |  |  |
| Results | | |  |  |  |  |  |
| Participants | 13* | (a) Report numbers of individuals at each stage of study—eg numbers potentially eligible, examined for eligibility, confirmed eligible, included in the study, completing follow-up, and analysed | 4919 patients initially; 33 patient deaths | 1912 patients initially; 103 patient deaths | 7713 patients initially; 807 patient deaths |  |  |
|  |  | (b) Give reasons for non-participation at each stage | Death | Death | Death |  |  |
|  |  | (c) Consider use of a flow diagram |  |  |  |  |  |
| Descriptive data | 14* | (a) Give characteristics of study participants (eg demographic, clinical, social) and information on exposures and potential confounders | See 6 | See 6 | See 6; For each diabetic patient, 5 non-diabetic patient were selected with matched age and gender |  |  |
|  |  | (b) Indicate number of participants with missing data for each variable of interest | Not reported | 6 removed due to missing data | Not reported |  |  |
|  |  | (c) Summarise follow-up time (eg, average and total amount) | 13 years | 10 years | 8 years |  |  |
| Outcome data | 15* | Report numbers of outcome events or summary measures over time | 10 outcomes | 12 outcomes | 9 outcomes |  |  |
| Main results | 16 | (*a*) Give unadjusted estimates and, if applicable, confounder-adjusted estimates and their precision (eg, 95% confidence interval). Make clear which confounders were adjusted for and why they were included | 33 patient deaths; SMR women 384% cf. 262% for men, cf. gen pop | 103 patient deaths; mean age of death 26.1 yr; overall SMR 4.0; | Annual mortality rates 8/1000 person years for Type I cf. 2.4/1000 without diabaetes. HR 3.7; Cardiovascular disease predominant cause of death |  |  |
|  |  | (*b*) Report category boundaries when continuous variables were categorized | Not reported | Not reported | Not reported |  |  |
|  |  | (*c*) If relevant, consider translating estimates of relative risk into absolute risk for a meaningful time period | Not reported | Not reported | Not reported |  |  |
| Other analyses | 17 | Report other analyses done—eg analyses of subgroups and interactions, and sensitivity analyses | Not reported | Not reported | Not reported |  |  |
| Discussion | | |  |  |  |  |  |
| Key results | 18 | Summarise key results with reference to study objectives | A significant excess mortality in this young cohort, without signs of long-term diabetic complications, occurred in a country with good access to public health care system, inexpensive for the patients. | We found a high death rate from violent deaths, especially in males, in whom it was the greatest single cause of death under 30 years of age. In the age group 30–44 years it was exceeded only by CVD as a cause of death. Although the death rate from violent deaths in the general population was high in young males, it did constitute a twofold increased risk of death in our cohort. | Despite advances in the care of patients with diabetes, mortality rates continue to be greatly elevated in those with type 1 diabetes in the UK. The greater elevation in risk in women than men with diabetes compared with the general population has persisted. The main cause of death in patients with type 1 diabetes was CVD, with greatly elevated relative risks compared with those without diabetes. |  |  |
| Limitations | 19 | Discuss limitations of the study, taking into account sources of potential bias or imprecision. Discuss both direction and magnitude of any potential bias | In our cohort it was not possible retrospectively to obtain data on levels of glycated haemoglobin in a standardized way for all patients and the values obtained could not confirm an association with intensified treatment. Such information would have been of great interest since there are indications that strict metabolic control might blunt the cathecholamine response to hypoglycaemia. | Not reported | A limitation of our study is that risk factor data are not complete in the database, and being complex to extract, we therefore did not carry out proper risk stratification or construct a much needed risk engine for identifying those patients with type 1 diabetes at greatest risk of specific causes of death. Although many GPs do report risk factor data, they were not required at the setup to provide this information. Furthermore, no centralised laboratories have been used, for example for the measurement of glycated haemoglobin. The care given by GPs to patients with type 1  diabetes, is however, typical of care received by these patients in general. |  |  |
| Interpretation | 20 | Give a cautious overall interpretation of results considering objectives, limitations, multiplicity of analyses, results from similar studies, and other relevant evidence | Death at onset of diabetes occurred mainly among very young subjects. This group may have unrecognized symptoms, a more rapid progression into diabetic ketoacidosis and coma, and treatment may also be more difficult. An inverse association of DKA with age has been reported; children below 5 years of age being approximately twice as likely to present with coma than older children.I6 In terms of preventable deaths, ketoacidosis among already diagnosed cases certainly belong to this group, and in this cohort two children with diabetes for several years arrived at hospital with acidosis and cerebral oedema. Furthermore, a 19-year old girl was found dead in bed with signs of ketoacidosis  (hyperglycaemia plus heavy ketonaemia) at autopsy. In her case suicide by omission of insulin injections cannot be excluded. | It has been claimed that the younger the patient is at the onset of type 1 diabetes, the shorter he lives [27]. This is contrary to the finding of the present study. The reason might be that in the past acute metabolic complications were responsible for the majority of deaths in the youngest age group. It seems that this cause of death, though still high, has decreased. A reason might be greater awareness among medical staff and parents, leading to earlier diagnosis and adequate management of acute complications. The present study shows that acute metabolic complications were the largest single cause of death under the age of 30 years. This is in keeping with a recent study from the UK [13]. | Increased mortality risks were found across all age-bands, but these were especially high in the younger patients with type 1 diabetes compared with subjects without diabetes, and especially in young women. Although we did not find a significantly higher risk ratio in women compared with men with type 1 diabetes vs those without, the sex difference was abolished in those with diabetes (p=0.3). This greater risk of mortality in young women with type 1 diabetes has been reported before [6,12], but remains poorly understood. With respect to cause-specific mortality, diabetes was associated with an increased HR for CVD deaths and other miscellaneous causes of death, but not cancer deaths. |  |  |
| Generalisability | 21 | Discuss the generalisability (external validity) of the study results | The present figures from Sweden are comparable with those reported for similar age groups from Finland” and Norway,12 and also to a smaller Swedish study.13 In comparison to a hospital based report from Sweden looking at death rates within 10 years of diabetes onset in young individuals who became diabetic before 1947, the present study shows a sevenfold decrease of early morta1ity. Such decreases by calendar time have also been shown in other countries. This decrease over time is probably dependent on the concomitant improvement and sophistication in overall community care, educational level, and hospital management in these countries. | A study from the USA (Allegheny  County, PA) based on type 1 diabetic patients diagnosed before 18 years between 1965 and 1979 reported a significant improvement in the survival rate between the cohort diagnosed during 1965–1969 and that diagnosed during 1975–1979 [9]. Comparison between Japan and Finland of the mortality rate of childhood-onset type 1 diabetes cases diagnosed between 1965 and 1979 was performed as of 1995 [8]. Finland showed no improving tendency when the mortality of the cases diagnosed between 1975 and 1979 was compared with that for 1965–1969. In Japan, a dramatic improvement was observed between 1969 and 1979. However, Podar et al. [14] report a further decline in the mortality of childhood-onset type 1diabetes in Finland, diagnosed during 1980–1994. | The prevalence of type 1 diabetes was 2.15/1,000 persons in 1992 in the GPRD, which is comparable with the prevalence rates reported by the International Diabetes Federation in 2000 for the UK, France, Germany and the Netherlands, respectively, of 3.4, 1.6, 1.0 and 2.6 per 1,000 persons (http://www.idf.org data accessed on 17 September 2003). In addition, similar (absolute) age-specific death rates were found for the nondiabetic control group in the GPRD in comparison with the Office for National Statistics 2001. Our results were similar to the results of the large Diabetes UK cohort study [12]. They found standardised mortality rates of, respectively, 4.0 (95% CI 3.6–4.4) and  2.7 (95% CI 2.5–2.9) in women and men, using data up to 1997. Our more recent data showed relative risks of 4.5 (95% CI 3.5–5.6) in women and 3.3 (95% CI 2.7–4.0) in men. This is a robust finding based on the two largest studies in the UK. |  |  |
| Other information | | |  |  |  |  |  |
| Funding | 22 | Give the source of funding and the role of the funders for the present study and, if applicable, for the original study on which the present article is based | Swedish Medical Research Council; Swedish Diabetes Association; Novo Nordisk Pharma, Sweden | Norwegian Diabetes Association; Diabaetes Research Centre | British Heart Foundation |  |  |

|  | Item No | Recommendation | Swerdlow & Jones, 1995 [39] | Waernbaum et al, 2006 [40] | Wibell et al, 2001 [41] |  |  |
| --- | --- | --- | --- | --- | --- | --- | --- |
| **Title and abstract** | 1 | (*a*) Indicate the study’s design with a commonly used term in the title or the abstract | Mortality during 25 years of follow-up of a cohort with diabetes | Excess mortality in incident cases of diabetes mellitus aged 15 to 34 years at diagnosis: a population-based study (DISS) in Sweden.[Erratum appears in Diabetologia. 2006 Jun;49(6):1457] | Increased mortality in diabetes during the first 10 years of the disease. A population-based study (DISS) in Swedish adults 15-34 years old at diagnosis |  |  |
|  |  | (*b*) Provide in the abstract an informative and balanced summary of what was done and what was found | BACKGROUND: Diabetes is one of the most common chronic diseases in Western populations. There have been few large published cohort studies of people with diabetes that have had more than 10 years of follow-up, and none other than the present one are in the UK. Such studies are important to understand the long-term fatal consequences of diabetes and their variation over time and between countries. METHODS: Cause-specific mortality was analysed in follow-up from 1966-1970 to December 1992 of 5783 members of the British Diabetic Association living in England and Wales during 1966-1970. Comparison was made with age-, sex- and calendar year-specific mortality by cause in the general population of England and Wales. RESULTS: During the follow-up 3399 (58.8%) subjects died. The relative risk of all-cause mortality in the cohort compared to the general population was 2.31 in women and 1.58 in men (both P < 0.001).Relative risks were greater for women than men at almost all ages and for each major diabetes-related cause of death. Absolute excess ('attributable') mortality rates were also greater in women than in men, except at ages < 50. Half the deaths in each sex were from circulatory diseases and only 3.4% were from renal disease. The relative risks of mortality for all-causes and circulatory diseases were particularly great at younger ages, but changed little with duration of follow-up. At ages < 40 the relative risks for all-causes were 3.75 in men and 5.51 in women and for ischaemic heart disease were 10.44 and 25.25 respectively (all P < 0.001). At these ages one-third of deaths were due to acute complications of diabetes, suicides and accidents, whereas at older ages these accounted for only 4% of deaths. CONCLUSIONS: The mortality rates at young ages in the cohort were around twice those in Sweden, Norway and Israel, suggesting that many of the deaths in England and Wales are preventable. The results also indicate a particular need for investigation and amelioration of cardiovascular risk factors in English and Welsh patients, especially women. | AIMS/HYPOTHESIS: The objective of the study was to analyse the mortality, survival and cause of death patterns in incident cases of diabetes in the 15-34-year age group that were reported to the nationwide prospective Diabetes Incidence Study in Sweden (DISS). MATERIALS AND METHODS: During the study period 1983-1999, 6,771 incident cases were reported. Identification of deaths was made by linking the records to the nationwide Cause of Death Register. RESULTS: With an average follow-up of 8.5 years, resulting in 59,231 person-years, 159 deaths were identified. Diabetes was reported as the underlying cause of death in 51 patients (32%), and as a contributing cause of death in another 42 patients (26%). The standardised mortality ratio (SMR) was significantly elevated (RR=2.4; 95% CI: 2.0-2.8). The SMR was higher for patients classified by the reporting physician as having type 2 diabetes at diagnosis than for those classified as type 1 diabetic (2.9 and 1.8, respectively). Survival analysis showed significant differences in survival curves between males and females (p=0.0003) as well as between cases with different types of diabetes (p=0.005). This pattern was also reflected in the Cox regression model showing significantly increased hazard for males vs females (p=0.0002), and for type 2 vs type 1 (p=0.015) when controlling for age. CONCLUSIONS/INTERPRETATION: This study shows a two-fold excess mortality in patients with type 1 diabetes and a three-fold excess mortality in patients with type 2 diabetes. Thus, despite advances in treatment, diabetes still carries an increased mortality in young adults, even in a country with a good economic and educational patient status and easy access to health care. | Objectives. To study. prospectively, in young adult patients, the mortality during the first years after the diagnosis of diabetes. Design. The Diabetes Incidence Study in Sweden (DISS) aims to register all incident cases aged 15-34 years. During a 10-year period all deaths were identified by record linkage to the national Cause of Death Registry. Subjects. During the period, 4097 new cases were registered and classified as type 1 diabetes (73%). type 2 (16%), secondary (2%) and unclassified (9%). The median follow-up was 5 years (21 001 person-years). Main outcome measures. Calculation of the standardized mortality ratio (SMR) and 95% confidence interval (CI). Evaluation of all deceased by scrutiny of clinical records, death certificates and autopsy protocols. Results. Fifty-eight patients died, corresponding to an SMR of 3.5 (CI = 2.7-4.5), which increased from 1.5 at 15-19 years to 4.1 at 30-34 years, SMR was 2.7 in primary diabetes: 2.3 (1.6-3.3) in type 1 and 4.1 (2.6-6.7) in type 2. In secondary diabetes, alcohol-associated pancreatitis a common cause, SMR was 32 (CI = 24-45). Evidence of alcohol or drug misuse, mental dysfunction or suicide was found in 40 of all 58 deceased cases. Less often, hypoglycaemia (n = 7) or hyperglycaemia-ketoacidosis (n = 11) was present at death. Unexplained 'dead in bed' was found once. Conclusions. In the investigated population-based cohort the early mortality was about threefold increased. Hypoglycaemia and ketoacidosis per se played a relatively small role compared with a heavy impact from social and mental dysfunction, and from careless use of alcohol or drugs. |  |  |
| Introduction | | |  |  |  |  |  |
| Background/rationale | 2 | Explain the scientific background and rationale for the investigation being reported | Diabetes is exceptional among chronic diseases in being a major cause of morbidity at virtually all ages. In childhood it is the second most prevalent chronic disease after asthma. By the age of 20 its cumulative incidence is the same as that for all cancers combined. It is important in young adulthood, and at older ages is one of the most common causes of death. Overall, the lifetime incidence in the UK is about 10%.' | Excess mortality of patients with diabetes mellitus has been shown in several studies [1–3]. In children below the age of 15 years at diagnosis, studies based on the Swedish Childhood Diabetes Register have shown significantly increased short-term mortality [4, 5]. Similar results have been observed in other countries, e.g. in the United Kingdom for children diagnosed before the age of 17 years [6]. However, few studies of mortality in young adults are available. | Diabetes mellitus is an important underlying or contributory cause of death. In the long term, the excess mortality in diabetes is mainly linked to cardiovascular disorders and, after a disease duration of 20 years, to diabetic nephropathy. During the last decades, progress in the management of diabetes and its complications has improved the outlook, particularly in young diabetic patients. Still, a considerably impaired  survival was recently described in young subjects with a disease duration of less than 20 years. |  |  |
| Objectives | 3 | State specific objectives, including any prespecified hypotheses | Since mortality in people with diabetes varies greatly by country and is changing over time, there is a need for more recent UK data on a large enough cohort to enable examination of risks by cause, age and sex, and hence to draw implications for clinical care. We present here data on the mortality of nearly 6000 people with diabetes in England and Wales identified in the late 1960s, who have now been followed for up to 26 years during which over 3000 deaths have occurred. | By linking the DISS records with the nationwide Cause of Death Registry, it is possible to identify deaths among the patients and obtain information on the date and cause of death. The aim of this study was to analyse the mortality, survival and cause of death patterns in incident cases of  diabetes aged 15–34 years in relation to some basic characteristics at diagnosis | In this study we aimed to evaluate the pattern of early mortality, and identify contributory factors, in young adult diabetic patients within 10 years of diagnosis. The follow-up comprised all incident cases of diabetes, aged 15±34 years, registered in Sweden from 1983 to 1992. The cohort was linked to the National Cause of Death Registry in order to identify all deceased patients and to compare the diabetic cohort with the general population. |  |  |
| Methods | | |  |  |  |  |  |
| Study design | 4 | Present key elements of study design early in the paper | Cohort of BDA patients, from Jan 1966-1970 – being followed up in 1992 to compare against gen pop, and within age, sex and calendar year specific mortality in England and Wales | By linking DISS with the Cause of Death Registry until 31 December 1999 information on date and cause of death  (underlying and contributing causes of death in terms of the International Classification of Diseases [ICD] classification) was received for 159 cases. The median follow-up time was 8.5 years (range: 0–17), representing 59,231 person-years. | The present investigation included all subjects reported to DISS until December 1992, a 10-year period. The background population, aged 15±34, was 2 336 300 individuals (1983), evenly distributed between the four 5-year age groups: 15±19 (607 700), 20±24 (548 400),  25±29 (569 500) and 30±34 (610 200) years. The size of these age groups varied little from year to year. |  |  |
| Setting | 5 | Describe the setting, locations, and relevant dates, including periods of recruitment, exposure, follow-up, and data collection | See 6 | See 6 | See 6 |  |  |
| Participants | 6 | (*a*) Give the eligibility criteria, and the sources and methods of selection of participants. Describe methods of follow-up | 5783 patients; UK; Any age; from 1966-1970; Member of British Diabetic Association; followed up in 31-Dec-1992 via National Health Service Register | 6771 patients; Sweden; 1983-1999; Diagnosis of Diabetes; 15-34 yrs old at time of diagnosis; participant of Diabetes Incidence Study in Sweden; followed up on 31-Dec-1999 via Swedish Cause of Death Registry | 4097 patients, Diagnosis of Diabetes; 15-34 yrs old at time of diagnosis; participant of Diabetes Incidence Study in Sweden; followed up on 31-Dec-1992 via Swedish Cause of Death Registry |  |  |
|  |  | (*b*) For matched studies, give matching criteria and number of exposed and unexposed | Not reported | Not reported | Not reported |  |  |
| Variables | 7 | Clearly define all outcomes, exposures, predictors, potential confounders, and effect modifiers. Give diagnostic criteria, if applicable | Age; Sex | Age; Sex | Age; Sex |  |  |
| Data sources/ measurement | 8* | For each variable of interest, give sources of data and details of methods of assessment (measurement). Describe comparability of assessment methods if there is more than one group | National Health Service Register | Swedish Cause of Death Registry | DISS and Cause of Death Registry |  |  |
| Bias | 9 | Describe any efforts to address potential sources of bias | Exclusion of patients with missing data points | Exclusion 19 patient due to missing values; 17 patient due to incomplete ID's | Exclusion of 17 patients due to being lost in follow up |  |  |
| Study size | 10 | Explain how the study size was arrived at | From cohort size | From cohort size | From cohort size |  |  |
| Quantitative variables | 11 | Explain how quantitative variables were handled in the analyses. If applicable, describe which groupings were chosen and why | Not reported | Not reported | Not reported |  |  |
| Statistical methods | 12 | (*a*) Describe all statistical methods, including those used to control for confounding | SMR; Poisson distribution; | SMR; Poisson distribution; Kaplan-Meier; log-rank test; Cox proportional hazard model | SMR; |  |  |
|  |  | (*b*) Describe any methods used to examine subgroups and interactions | Not reported | Not reported | Not reported |  |  |
|  |  | (*c*) Explain how missing data were addressed | Patients excluded from study | Patients excluded from study | Patients excluded from study |  |  |
|  |  | (*d*) If applicable, explain how loss to follow-up was addressed | Not reported | Not reported | Not reported |  |  |
|  |  | (*e*) Describe any sensitivity analyses | Not reported | Not reported | Not reported |  |  |
| Results | | |  |  |  |  |  |
| Participants | 13* | (a) Report numbers of individuals at each stage of study—eg numbers potentially eligible, examined for eligibility, confirmed eligible, included in the study, completing follow-up, and analysed | 5783 patients initially; 3399 patient deaths | 6771 patients initially; 159 patient deaths | 4097 patients initially; 58 patient deaths |  |  |
|  |  | (b) Give reasons for non-participation at each stage | Not reported | 28 patient with secondary diabetes excluded | Not reported |  |  |
|  |  | (c) Consider use of a flow diagram |  |  |  |  |  |
| Descriptive data | 14* | (a) Give characteristics of study participants (eg demographic, clinical, social) and information on exposures and potential confounders | See 6 | See 6 | See 6 |  |  |
|  |  | (b) Indicate number of participants with missing data for each variable of interest | Not reported | Not reported | Not reported |  |  |
|  |  | (c) Summarise follow-up time (eg, average and total amount) | 4 year study period; 25 year follow up | 15 year study period | 10 years |  |  |
| Outcome data | 15* | Report numbers of outcome events or summary measures over time | 8 outcomes | 10 outcomes | 10 outcomes |  |  |
| Main results | 16 | (*a*) Give unadjusted estimates and, if applicable, confounder-adjusted estimates and their precision (eg, 95% confidence interval). Make clear which confounders were adjusted for and why they were included | 3399 patient deaths; all-cause mortality relative risk cf. gen. pop 2.31 in women, 1.58 in men; | 159 patient deaths; SMR 2.4; diabetes as underlying cause of death in 51 patient | 58 patient deaths; SMR 3.5; SMR 2.7 in Type I - 4.1 in Type II; Alcohol/drug misuse/mental dysfunction/suicide was linked to 40/58 deaths |  |  |
|  |  | (*b*) Report category boundaries when continuous variables were categorized | Not reported | Not reported | Not reported |  |  |
|  |  | (*c*) If relevant, consider translating estimates of relative risk into absolute risk for a meaningful time period | Not reported | Not reported | Not reported |  |  |
| Other analyses | 17 | Report other analyses done—eg analyses of subgroups and interactions, and sensitivity analyses | Not reported | Not reported | Not reported |  |  |
| Discussion | | |  |  |  |  |  |
| Key results | 18 | Summarise key results with reference to study objectives | There were significantly raised  SMR (at least P < 0.05) in each sex for deaths certified to diabetes, other disorders of pancreatic internal secretion, ischaemic heart disease, cerebrovascular disease, and pneumonia; in males for non-pancreatic endocrine and metabolic disorders; and in females for hypertensive heart disease, heart disease other than hypertensive or ischaemic, vascular disease other than of the cardiac or cerebral vasculature, digestive diseases, diseases of the urinary system (for which risk was also raised, but not significantly, in males), and injury and poisoning, especially accidental falls. | In this study the SMR for types 1 and 2 diabetes were 1.8 and 2.9 based on a mean follow-up time of 8.5 years. Thus this study confirms the earlier findings of a two-fold excess mortality for incident cases with type 1 diabetes and a threefold excess mortality for incident cases with type 2 diabetes in patients between 15 and 34 years at diagnosis. | A SMR of 3.5, in the diabetes cohort. The incidence of insulin-dependent type 1 diabetes is high in Sweden and virtually all newly diagnosed young patients are referred to public hospitals or special diabetes clinics, all of which report to DISS. Type 1 and type 2 patients contributed to the overall threefold increased mortality. However, the subgroup of subjects less than 20 years of age and the small groups of female type 1 and type 2 patients showed small elevations of SMR which were not significant. In secondary diabetes, accounting for 25% of all deaths, a 30-fold increase in SMR was found. |  |  |
| Limitations | 19 | Discuss limitations of the study, taking into account sources of potential bias or imprecision. Discuss both direction and magnitude of any potential bias | The BDA members are a self-selected group who might be more health-conscious and careful in their diabetes control, diet, and clinic attendance, than non-members, and therefore their mortality might also be less. Although we do not have direct information on this for the study cohort, 27% of current BDA members are from social grades A and B (professional and managerial) compared to 16% of the national population (Murphy M, personal communication), implying that their experience may indeed be somewhat better than average. Potential for selection applies particularly to those with NIDDM, for whom a smaller proportion of all affected people in the country are members (currently estimated at 12%) and less to people with IDDM (of whom it is currently estimated 38% are members). | With the knowledge that diabetes in many cases is not mentioned on the death certificate and patients at risk are often lost to follow-up before the time of death, it is evident that a mortality study in diabetes must be prospective and population-based. | Death certificates are often written with limited access to clinical data and, as in other studies, the presence of diabetes was not mentioned on the certificate in 50% of the cases. |  |  |
| Interpretation | 20 | Give a cautious overall interpretation of results considering objectives, limitations, multiplicity of analyses, results from similar studies, and other relevant evidence | In our data as in previous studies, the all-cause SMR decreased with attained age beyond about age 40. This finding is not simply a reflection of the greater relative risk of death for people with IDDM (generally young) than NIDDM (generally older), since it occurred steadily throughout the age range, and has been found separately in insulin-treated and non-insulin treated subjects. There is limited previous information on cause-specific SMR by age, but the available data generally accord with the present results that ischaemic heart disease and cerebro-vascular disease SMR decrease with age, although one study did not find this for ischaemic heart disease after adjusting for other risk factors. | The fact that incident cases of type 2 diabetes can have carried the disease for several years before they are diagnosed  might explain why the SMR for patients classified as having type 2 diabetes at diagnosis are slightly higher than the SMRs for cases with type 1 diabetes at diagnosis  (SMR=2.9 and 1.8, respectively). In addition, patients with type 2 diabetes had a significantly higher BMI than the patients with type 1 diabetes. High BMI is a known risk factor for many circulatory diseases | In an affluent society, with access to medical  care, young adults with diabetes may already have an increased mortality rate during the first years after diagnosis. The risk appears to be largely determined by the coexistence of detrimental psychosocial factors. Whether it is possible to almost eliminate the excess mortality, as reported by Modan et al. [10], might depend on cultural factors, religion and family structure. In a Swedish population, most conspicuous in men, we found that alcohol and/or drug abuse contributed to the mortality. |  |  |
| Generalisability | 21 | Discuss the generalisability (external validity) of the study results | In previous British data the great majority of people with diabetes aged 3=50 had NIDDM, whereas among current BDA members this is less striking: 34% of those aged 40-59 and 58% at ages 3=60 have NIDDM, so our cohort entering at these ages may be similarly mixed, and hence less representative of prognosis in those with diabetes in general. | Thus, the difference in hazard for men with  diabetes compared with women with diabetes only reflects the increased general hazard in the Swedish population for men to die compared with women. This could also have been the case in a study in Denmark of a cohort of 906 patients diagnosed before 1943 at an age <31 years. These patients were followed-up and it was found that long-term survival among patients who had been aged 16–30 years at diagnosis was significantly better among women than men [26]. Similarly, in a study in Pittsburgh of 1,894 patients diagnosed between 1950 and 1981 at an age <17 years, SMR values of 11.5 and 5.4, respectively, were found in men and women [27]. In contrast, a study in southern Wisconsin of 1,210 cases with diabetes diagnosed before the age of 30 years arrived at SMRs of 6.8 and 8.9, respectively, for men and women [28]. | We found, with borderline significance, an  approximately twofold increased suicide rate in  diabetes, similar to observations from USA and  Norway [24,26] but not in accordance with Deckert et al. [3]. The reported frequency of accidents and suicide in diabetic patients, more common in Finland than in Japan, certainly seems to vary between countries [9]. Diabetes, in the absence of complications, has been claimed to affect social life surprisingly little [27]. Nevertheless, not least when recently acquired, diabetes might well be a life event able to induce an increased suicidal risk. |  |  |
| Other information | | |  |  |  |  |  |
| Funding | 22 | Give the source of funding and the role of the funders for the present study and, if applicable, for the original study on which the present article is based | Medical Research Council | Swedish Medical Research Council; Swedish Diabetes Association; Novo Nordisk Foundation; Juvenile Diabetes Foundation-Wallenberge Diabetes Research Programme | NIH grant DK42634; Jubenile Diabetes Foundation-Wallenberg Diabetes Research Program; Swedish Medical Research Council; Swedish Diabetes Association |  |  |

**Figure S1:** Cumulative meta-analysis by median study date (<=1970, 1971-1980, 1981-1990, >1990). Horizontal bars and circles widths denote 95% CIs, and box sizes indicate relative weight in the analysis

**Figure S2:** Trim-and-fill analysis of the included estimates. No trimming of data was performed, suggesting no evidence of publication bias

Test for heterogeneity: Q=2193.113 on 87 degrees of freedom (p<0.01).
Moment-based estimate of between studies variance = 0.246

* However, the funnel plot does not take into account the between study heterogeneity (between studies variance = 0.246). The considerable amount of between-study heterogeneity suggests the true funnel plot (if considering both within-study and between-study heterogeneity) should be wider.
